# Impact of the COVID-19 pandemic on agricultural production, livelihoods, and food security in India: baseline results of a phone survey

**DOI:** 10.1007/s12571-021-01164-w

**Published:** 2021-05-13

**Authors:** Lindsay M. Jaacks, Divya Veluguri, Rajesh Serupally, Aditi Roy, Poornima Prabhakaran, GV Ramanjaneyulu

**Affiliations:** 1 Global Academy of Agriculture and Food Security, The University of Edinburgh, Easter Bush Campus, Midlothian, EH25 9RG UK; 2 Harvard T.H. Chan School of Public Health, Harvard University, Boston, MA USA; 3 Public Health Foundation of India, New Delhi, India; 4 Centre for Chronic Disease Control, New Delhi, India; 5 Centre for Sustainable Agriculture, Hyderabad, India

**Keywords:** Food production, Farmers, Food security, COVID-19, Pandemic, South Asia

## Abstract

The aim of this study was to evaluate the impact of the COVID-19 lockdown on agricultural production, livelihoods, food security, and dietary diversity in India. Phone interview surveys were conducted by trained enumerators across 12 states and 200 districts in India from 3 to 15 May 2020. A total of 1437 farmers completed the survey (94% male; 28% 30–39 years old; 38% with secondary schooling). About one in ten farmers (11%) did not harvest in the past month with primary reasons cited being unfavorable weather (37%) and lockdown-related reasons (24%). A total of 63% of farmers harvested in the past month (primarily wheat and vegetables), but only 44% had sold their crop; 12% were still trying to sell their crop, and 39% had stored their crop, with more than half (55%) reporting lockdown-related issues as the reason for storing. Seventy-nine percent of households with wage-workers witnessed a decline in wages in the past month and 49% of households with incomes from livestock witnessed a decline. Landless farmers were about 10 times more likely to skip a meal as compared to large farmers (18% versus 2%), but a majority reported receiving extra food rations from the government. Nearly all farmers reported consuming staple grains daily in the past week (97%), 63% consumed dairy daily, 40% vegetables daily, 26% pulses daily, and 7% fruit daily. These values are much lower than reported previously for farmers in India around this time of year before COVID-19: 94–95% dairy daily, 57–58% pulses daily, 64–65% vegetables daily, and 42–43% fruit daily. In conclusion, we found that the COVID-19 lockdown in India has primarily impacted farmers’ ability to sell their crops and livestock products and decreased daily wages and dietary diversity.

**Supplementary Information:**

The online version contains supplementary material available at 10.1007/s12571-021-01164-w.

## Introduction

In response to the coronavirus disease 2019 (COVID-19) pandemic, the Government of India imposed the largest lockdown in history: 1.3 billion people were required to shelter in place from 25 March to 8 June 2020. There is no doubt that this lockdown disproportionately affected the poor and daily wage earners (Ahmed Mushfiq Mobarak 2020; Cash and Patel 2020), including rural farmers. Even before the COVID-19 crisis, the low incomes of farmers were a critical issue in India, with the Government of India setting a goal to double farmers’ income by 2022. A survey conducted in 2016–17 found that the average income of farming households stands at 8931 INR (~$118), of which 35% comes from cultivation, 34% from wage earnings, and 8% from livestock (NABARD 2018). A shortfall in any of these sources of income could significantly impact farmer households. However, the magnitude of the impact of the COVID-19 lockdown on farmers’ agricultural production, experience of food insecurity, income from livestock, and daily wages is still largely unknown. Understanding this impact has important implications for preparing for upcoming agricultural seasons, informing the targeted provision of emergency food rations to those most in need, and re-building a more resilient, sustainable, and equitable agri-food system.

Six previous studies relating to agricultural production, livelihoods, and food security during the COVID-19 lockdown in India have recently been reported (Acharya 2020; Azim Premji University 2020; Ceballos et al. 2020; Harris et al. 2020; Totapally et al. 2020; Vikas Anvesh Foundation 2020). A survey focused on the financial impact of the lockdown and coverage of government benefits was conducted in 6915 households identified as Below Poverty Line between 7 and 9 of April (Totapally et al. 2020). They reported that 43% of men and 48% of women worried about getting daily essentials such as groceries, and 43% of those who were eligible for free food rations through the Public Distribution System (PDS) did not receive them (Totapally et al. 2020). The primary reasons cited were stocks not being available and restrictive lockdown rules (Totapally et al. 2020). A second survey, conducted between 27 April and 2 May (5162 respondents), more explicitly evaluated food security (Vikas Anvesh Foundation 2020). Half of surveyed households reported a reduction in the number of meals consumed and two-thirds reported a reduction in the number of items in a meal despite a majority (84%) having received food rations through PDS (Vikas Anvesh Foundation 2020). A third study, conducted in mid-April (3970 respondents including 331 farmers), had similar results: 70% of rural households reported consuming “less food than before” despite 91% having received PDS support and 53% receiving a cash transfer from the government (Azim Premji University 2020). Among the 331 farmers, 37% reported being unable to harvest their crop and 15% reported being unable to sell their harvest due to the lockdown (Azim Premji University 2020). A study in two states (1694 respondents) found that 48% of households in Bihar and 32% of households in Uttar Pradesh reported shortages of food items during the lockdown (Acharya 2020). A fifth study reported findings from surveys of participants in World Vegetable Center’s programs in four states (448 respondents): over 80% of farmers reported some decline in sales, 90% reported a drop in farm income, and 62% reported disruptions to their habitual food consumption including reduced access to nutrient-dense foods such as fruits and animal-source products (Harris et al. 2020). Finally, a study of 1515 smallholder farmers conducted between early-April and mid-May found that in Haryana, 41% spent more on harvesting their wheat this year and 61% stored their harvest, and in Odisha, 80% spent more on harvesting their black gram this year and 74% stored their harvest (Ceballos et al. 2020). They also reported little impact of the lockdown on food insecurity citing short value chains, homestead gardens, and a well-functioning PDS as potential mitigation factors (Ceballos et al. 2020).

In sum, these previous studies have highlighted potentially major impacts on agricultural production and food security as a result of the COVID-19 lockdown in India. However, only two of these studies focused explicitly on agricultural households (Ceballos et al. 2020; Harris et al. 2020), and were limited to a few states and production systems, thus not generalizable to all of India. The aim of this study was to evaluate the impact of the COVID-19 lockdown on agricultural production, livelihoods, food security, and dietary diversity in India – including evaluating differences by state and crop type. This level of detail is needed to inform specific policy and programmatic recommendations that address the adverse effects of the lockdown on agricultural households.

## Methods

### Survey sample

**Fig. 1:**
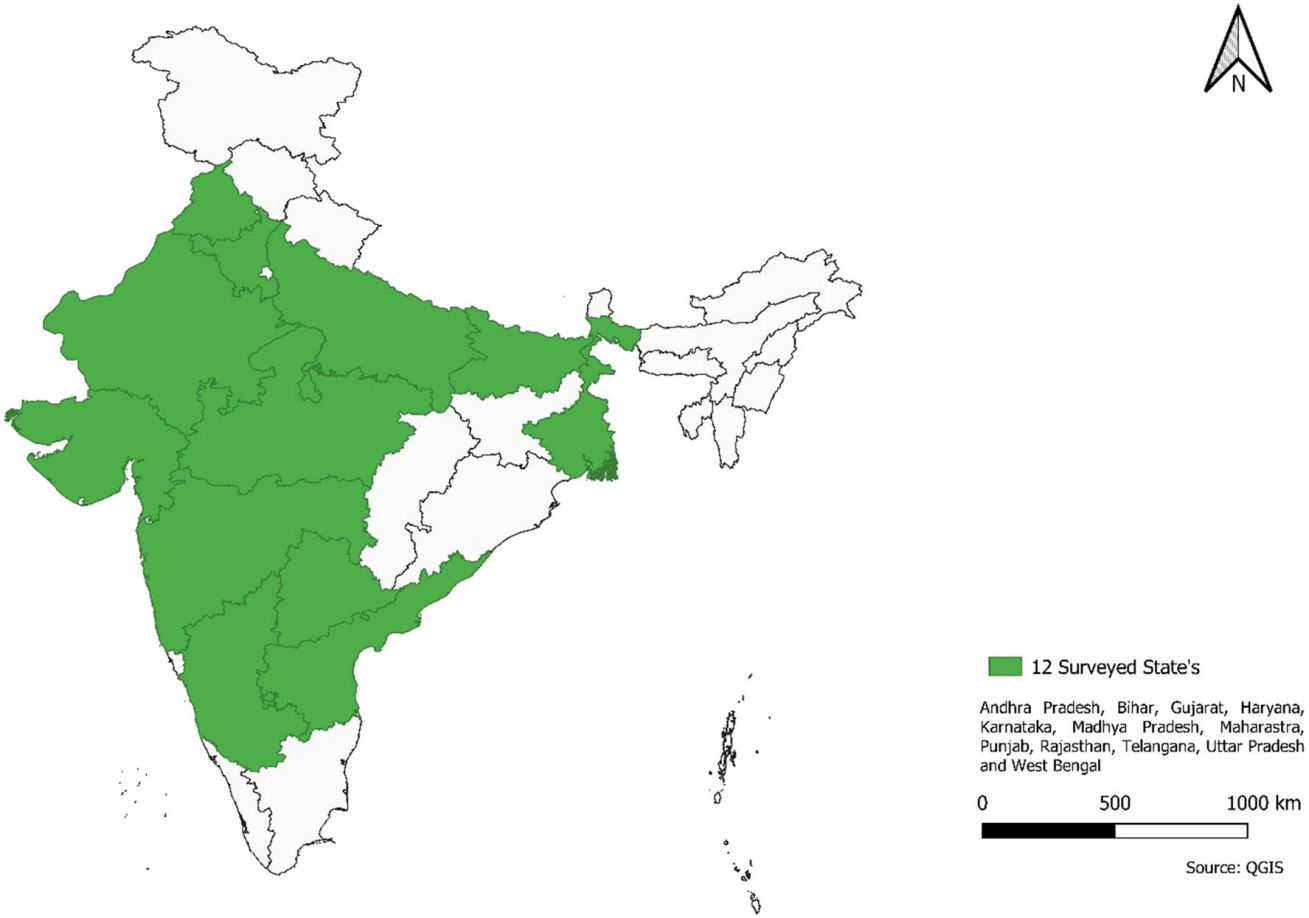
National coverage of survey in 12 states and 200 districts

Participants were recruited from the top 12 agricultural producing states in India according to total acres of food grains (e.g., rice, wheat, maize, and pulses), oil seeds, and cash crops (e.g., sugarcane and cotton) planted in 2018 (Department of Agriculture Cooperation and Farmers Welfare 2018). These states included (in alphabetical order): Andhra Pradesh, Bihar, Gujarat, Haryana, Karnataka, Madhya Pradesh, Maharashtra, Punjab, Rajasthan, Telangana, Uttar Pradesh, and West Bengal (Fig. 1). Across these 12 states, participants came from 200 districts. Eligibility criteria included being an adult aged ≥18 years and belonging to an agricultural household defined as any one or more of the following: owning land, harvesting a crop in the past month regardless of land ownership, earning a daily wage or contract-based wage from agricultural work, and earning an income from livestock or fishing. Thus, respondents that did not own land, did not harvest any land in the past month, did not have an income from wages (daily wages or contract-based work), and who did not have an income from livestock or fishing were not considered farmers or wage laborers and excluded from the analysis (*n* = 19).

The initial seed sample was recruited via the pre-existing networks of the Centre for Sustainable Agriculture and 12 other civil society organizations implementing agroecology programs or broadly working in rural development (e.g., education; women’s empowerment; livelihoods; water, sanitation and hygiene; food and nutrition). The civil society organizations were primarily working in Andhra Pradesh, Bihar, Gujarat (three organizations), Karnataka (three organizations), Madhya Pradesh, Punjab, Rajasthan, Telangana, Uttar Pradesh (two organizations), and West Bengal. In Haryana and Maharashtra, we relied more on personal contacts (e.g., an informal network of public policy professionals). We then snowball sampled, calling up to four additional farmers per respondent. In order to increase the diversity of the survey sample, interviewers were trained to specifically request contact information from all categories of agricultural households: farmers and wage laborers, large farms and small/marginal farms, men and women, and inclusive of livestock and fishing. This led to the final sample of 1437 households from across 200 districts in 12 states.

### Ethics

This study was reviewed and approved by the Harvard T.H. Chan School of Public Health Institutional Review Board (Protocol #: IRB20-0689) and the Public Health Foundation of India Institutional Ethics Committee (Protocol #: TRC-IEC 438/20). Verbal informed consent was obtained from all respondents.

### Data collection

All surveys were conducted via phone interview between 3 May and 15 May 2020 and responses were entered directly into Qualtrics (Qualtrics, Provo, Utah). The survey (**Supplementary Material**) took approximately 15 min to complete for households without a harvest in the past month and 30 min for households with a harvest. The survey was translated into the following eight languages (translations are available upon request): Hindi, Telugu, Marathi, Bengali, Rajasthani, Punjabi, Gujarati, and Kannada. All enumerators completed a 3-h training on implementing the survey tool prior to commencing data collection. The average response rate across states was 78.4%.

With regard to specific components of the survey, commercial agricultural production questions were similar to those asked in routine government surveys (e.g., Agricultural Census and Input Survey), and were adapted in consultation with agricultural experts and based on pilot testing. For questions that referred to the “previous season,” this was defined as January/February 2020 (pre-COVID-19) for crops harvested more than once a year (e.g., vegetables) and Rabi 2019 for crops harvested once a year (e.g., wheat). For all crops, the cost of transport in the past month was compared to what the same transport would have cost in January/February 2020. Land area owned and harvested were reported in local units and converted into hectares (ha) using state-specific conversions when applicable (**Supplementary Table** **1**). Four farm size categories were defined according to land ownership as landless (0 ha), small/marginal farms (0.01-2.00 ha), medium farms (2.01-4.00 ha), and large farms (>4.00 ha) (Department of Agriculture 2020). While some pre-specified options were provided for questions on reasons for not harvesting, for changes in cost to harvest, for yield loss, for storing, and for how the lockdown has impacted their ability to prepare for the sowing season, open-ended text entries were also permitted. These entries were reviewed and categorized for analysis.

All participants were asked if they or anyone in their household worked for wages, including day wages and contract-based wages. If yes, they were then asked how many people in the household worked for wages, and for each person who worked for wages, what their earnings were in the past month and what their earnings were at this time last year. We summed the total wages of all wage-working individuals in the household for analysis, and calculated the percent change in a household’s wages comparing the past month to this time last year. Based on this percent change, we categorized participants as having declines, increases, or no change in household wages. INR to USD conversions were made using the World Bank’s Global Economic Monitor exchange rate for 2020 M06 (75.7) (Global Economic Monitor 2020). Livestock ownership was categorized as “cows, buffalo, oxen, and bulls,” “poultry,” and “goats and sheep.” One respondent reported owning a camel and one owning a pig; given the small number, these were excluded from the livestock ownership analysis.

Food security was assessed using three questions from the Food and Agriculture Organization’s (FAO) Food Insecurity Experience Scale (FIES) (Ballard et al. 2013; Cafiero et al. 2018): in the past month, was there a time when you or others in your household (1) worried you would run out of food, (2) skipped a meal, or (3) went without eating for a whole day. Diet was assessed using questions from the FAO’s Minimum Dietary Diversity score for women (MDD-W) (FAO 2016), which has been used in previous studies of rural adult women and men in India (Singh et al. 2020). Specifically, eight of the ten MDD-W food groups were included: (1) starchy staples (rice, wheat, and potatoes), (2) pulses, (3) nuts, (4) vegetables, (5) fruits, (6) dairy, (7) eggs, and (8) fleshy foods (meat, poultry, and fish). Vegetables and fruits were not divided further into dark green leafy vegetables and vitamin A-rich fruits and vegetables versus other vegetables and fruits because we were conducting phone interviews and had to simplify the survey as much as possible to maximize participant engagement and data quality. Those who consumed a food group every day in the past week were assigned a value of “1” and those who did not were assigned a value of “0.” Values were then summed across the eight food groups such that the dietary diversity score ranged from 0 to 8 with 8 representing maximum dietary diversity. While the MDD-W typically only captures foods consumed in the past 24 h, we were interested in also capturing episodic consumption of nutrient-dense food groups such as fleshy foods, and so chose a recall period of 1 week.

### Statistical analysis

Data cleaning, management, and analysis were conducted using SAS version 9.4 (SAS Institute, Cary, North Carolina) and Stata release 16 (StataCorp LLC, College Station, Texas). Less than 5% of data were missing for all variables except caste (27% missing – asked during a follow-up survey), change in transport cost (53% missing), and awareness of government support measures for agriculture during the lockdown (57% missing), which were added partway through the baseline survey (**Supplementary Table** **2**). Descriptive statistics were used to summarize demographic characteristics (state, age, gender, household size, and caste), educational attainment, agricultural production (crop type, harvest, what was done with the harvest, and sowing), harvest cost, transport cost, government support programs, self-reported reasons for not harvesting, storing the harvest, higher harvest costs, lower yields, and concern over the upcoming sowing season, wages, livestock income, food insecurity, and dietary diversity, overall and by farm size. We also presented key outcomes by state, crop type, and caste. We tested for differences in these characteristics according to farm size, state, crop type, and caste using chi-square tests (for binary and categorical variables) and analysis of variance (for continuous variables). *P* values less than 0.05 were considered statistically significant. In order to provide further insight into these findings, we explored the association between receipt of cash transfers, declines in wages, and declines in livestock income with the harvest, sale, storage, and sowing of crops.

## Results

The average age of participants was 41.9 years (range: 18 to 85), 28% were between the ages of 30 and 39, and 94% were male (Table 1). Nearly one-third of participants reported having graduate degrees or above. Land ownership was, on average, 3.38 ha ranging from 0 to 263 ha (mean excluding two outliers with land ownership >100 ha was 3.13 ha); 51% of participants were small/marginal farmers. The four states with the greatest proportion of small/marginal farmers were: Bihar, Gujarat, Uttar Pradesh, and West Bengal (**Supplementary Table** **3**). Punjab and Rajasthan had the greatest proportion of large farmers. Landless farmers and small/marginal farmers were significantly more likely to be female, have no formal schooling, younger, and self-report belonging to a Scheduled Caste/Tribe or Other Backward Caste. Large farmers were significantly more likely to have households with 6 people or more.

**Table 1:** Demographic and socioeconomic characteristics of participants from agricultural households across 12 states and 200 districts in India during the national COVID-19 lockdown, according to farm size

Characteristic	Total (*n* = 1437)		Farm Size^*^								*P* value^†^
			Landless (*n* = 88)		Small/Marginal (*n* = 722)		Medium (*n* = 286)		Large (*n* = 318)		
State											
Andhra Pradesh	10%	(149)	28%	(25)	9%	(63)	14%	(39)	7%	(21)	<0.0001
Bihar	8%	(110)	5%	(4)	9%	(66)	6%	(18)	7%	(22)	
Gujarat	6%	(88)	1%	(1)	8%	(55)	7%	(19)	4%	(12)	
Haryana	6%	(83)	18%	(16)	3%	(25)	7%	(21)	6%	(19)	
Karnataka	7%	(100)	2%	(2)	8%	(58)	7%	(20)	6%	(19)	
Madhya Pradesh	10%	(149)	22%	(19)	10%	(70)	10%	(29)	9%	(29)	
Maharashtra	4%	(54)	3%	(3)	3%	(22)	3%	(8)	5%	(15)	
Punjab	11%	(161)	0%	(0)	5%	(37)	13%	(37)	27%	(85)	
Rajasthan	9%	(131)	5%	(4)	7%	(51)	9%	(26)	16%	(50)	
Telangana	13%	(180)	1%	(1)	14%	(101)	17%	(49)	9%	(29)	
Uttar Pradesh	8%	(109)	10%	(9)	9%	(66)	5%	(14)	4%	(12)	
West Bengal	9%	(123)	5%	(4)	15%	(108)	2%	(6)	2%	(5)	
Gender											
Male	94%	(1345)	81%	(71)	93%	(671)	97%	(277)	97%	(308)	<0.0001
Female	6%	(92)	19%	(17)	7%	(51)	3%	(9)	3%	(10)	
Age											
<30	16%	(224)	26%	(23)	15%	(111)	15%	(42)	15%	(46)	0.005
30–39	28%	(397)	34%	(30)	28%	(202)	26%	(74)	26%	(82)	
40–49	28%	(402)	15%	(13)	29%	(209)	30%	(86)	27%	(86)	
50–59	18%	(256)	21%	(18)	18%	(128)	15%	(42)	21%	(65)	
60+	10%	(149)	3%	(3)	9%	(68)	15%	(42)	11%	(36)	
Household size											
1–2 people	3%	(45)	9%	(8)	3%	(20)	3%	(9)	3%	(8)	0.001
3 people	7%	(95)	8%	(7)	8%	(54)	5%	(14)	6%	(20)	
4 people	24%	(341)	20%	(18)	26%	(190)	22%	(63)	21%	(66)	
5 people	20%	(281)	30%	(26)	20%	(144)	19%	(55)	16%	(50)	
6 or more people	47%	(668)	33%	(29)	43%	(312)	50%	(143)	54%	(172)	
Educational attainment											
No formal schooling	9%	(131)	27%	(24)	9%	(66)	5%	(15)	7%	(23)	<0.0001
Primary school	23%	(336)	33%	(29)	26%	(185)	23%	(66)	14%	(45)	
Secondary school	38%	(547)	25%	(22)	39%	(282)	40%	(114)	39%	(124)	
Grad/Post grad/Professional	29%	(418)	15%	(13)	26%	(189)	31%	(89)	39%	(125)	
Caste											
Scheduled Caste/Tribe	24%	(246)	45%	(29)	26%	(142)	21%	(41)	13%	(29)	<0.0001
Other Backward Caste	38%	(398)	17%	(11)	45%	(245)	35%	(69)	30%	(69)	
Other/No answer	38%	(401)	38%	(24)	28%	(154)	45%	(89)	57%	(131)	
Land ownership, ha^‡^	3.13	(5.00)	0.00	(0.00)	0.88	(0.52)	2.63	(0.55)	9.59	(7.38)	<0.0001

Values are percent (n) or mean (SD)

Abbreviations: ha, hectares

^*^Defined according to land ownership as landless (0 ha), small/marginal farms (0.01-2.00 ha), medium farms (2.01-4.00 ha), and large farms (>4.00 ha)

^†^P value from chi-square test (binary and categorical variables) or analysis of variance (continuous variables) comparing characteristics across farm sizes

^‡^Excludes *n*=2 farmers owning >100 ha of land

**Fig. 2:**
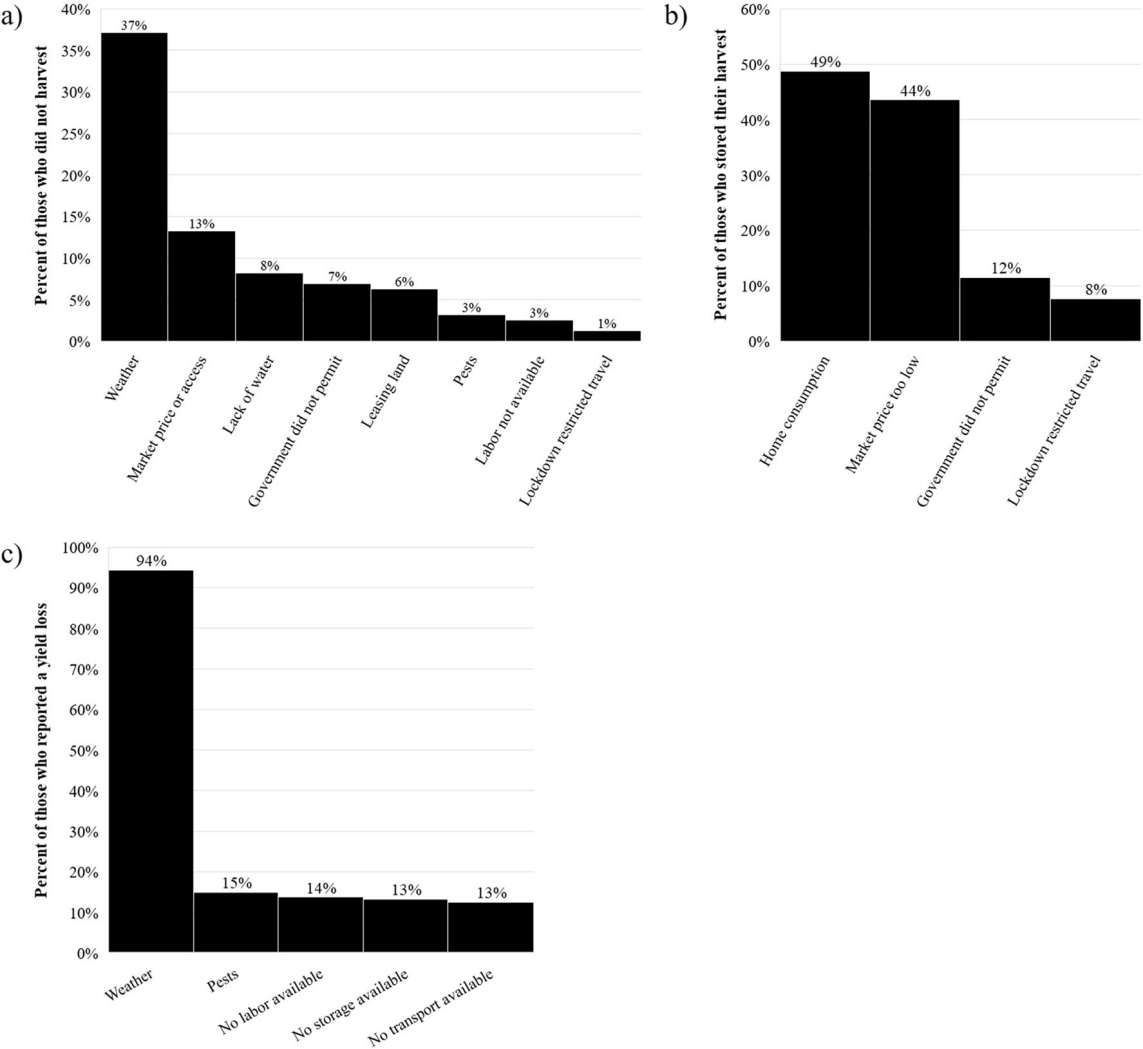
Reasons reported for (**a**) not harvesting in the past month (*n* = 159), (**b**) storing harvest in the past month (*n* = 312), and (**c**) yield losses in the past month (*n* = 423) in participants from agricultural households across 12 states and 200 districts in India during the national COVID-19 lockdown

Nearly two-thirds (63%) of participants had harvested in the past month (Table 2), and of those, 78% had harvested the same crop in the previous season. A total of 11% of participants did not harvest in the past month; Karnataka and Telangana had the greatest proportion reporting not harvesting in the past month (**Supplementary Table** **3**). While 37% of those who did not harvest reported weather-related reasons, many reported that it was due to a lockdown-related issue, including low market price or markets being closed, government restrictions on travel, and labor not being available (Fig. 2a).

**Table 2:** Agricultural production in agricultural households across 12 states and 200 districts in India during the national COVID-19 lockdown, according to farm size

Characteristic	Total (*n*=1437)		Farm Size^*^								*P* value^†^
			Landless (*n*=88)		Small/Marginal (*n*=722)		Medium (*n*=286)		Large (*n*=318)		
Harvested in past month											
Out of season	25%	(351)	31%	(15)	29%	(209)	26%	(75)	14%	(46)	<0.0001
Yes	63%	(884)	48%	(23)	58%	(421)	67%	(191)	77%	(245)	
No	11%	(159)	21%	(10)	13%	(92)	7%	(19)	8%	(27)	
Primary crop harvested in past month											
Wheat	60%	(532)	65%	(15)	52%	(220)	63%	(121)	71%	(175)	<0.0001
Vegetables	15%	(134)	4%	(1)	21%	(88)	14%	(26)	7%	(17)	
Pulses	4%	(39)	4%	(1)	5%	(22)	2%	(4)	5%	(12)	
Rice paddy	3%	(30)	4%	(1)	4%	(17)	2%	(4)	3%	(8)	
Maize	3%	(30)	9%	(2)	4%	(16)	4%	(7)	2%	(5)	
Other^‡^	14%	(120)	13%	(3)	14%	(58)	15%	(29)	12%	(29)	
What was done with the harvest in past month											
Sold it	44%	(389)	36%	(8)	33%	(141)	55%	(102)	55%	(134)	<0.0001
Stored it	39%	(344)	23%	(5)	46%	(193)	33%	(62)	34%	(84)	
Trying to sell it	12%	(108)	36%	(8)	16%	(67)	8%	(15)	7%	(18)	
Not yet decided	2%	(21)	0%	(0)	3%	(12)	2%	(3)	2%	(6)	
Wasted	2%	(17)	5%	(1)	2%	(8)	3%	(5)	1%	(3)	
Change in land harvested^‡^											
Decrease	13%	(89)	20%	(4)	17%	(55)	8%	(11)	10%	(19)	0.009
Increase	16%	(107)	10%	(2)	12%	(38)	18%	(27)	21%	(40)	
No change	71%	(490)	70%	(14)	71%	(228)	74%	(108)	70%	(136)	
Yield loss^‡^											
Yes	62%	(423)	55%	(11)	66%	(212)	58%	(83)	60%	(117)	0.26
No	38%	(260)	45%	(9)	34%	(109)	42%	(61)	40%	(77)	
Change in cost to harvest^§^											
Higher	53%	(367)	25%	(5)	60%	(193)	47%	(68)	51%	(100)	<0.0001
Lower	21%	(144)	50%	(10)	21%	(69)	18%	(26)	20%	(39)	
Same	26%	(175)	25%	(5)	18%	(59)	36%	(52)	29%	(56)	
Change in transport cost^§#^											
Higher	43%	(180)	14%	(1)	35%	(54)	46%	(51)	50%	(73)	0.03
Lower	2%	(10)	14%	(1)	2%	(3)	2%	(2)	3%	(4)	
Same	55%	(230)	71%	(5)	63%	(98)	52%	(58)	47%	(68)	
Lockdown impacted ability to sow for upcoming season											
Yes	55%	(752)	24%	(18)	52%	(360)	60%	(162)	68%	(206)	<0.0001
No	45%	(605)	76%	(56)	48%	(333)	40%	(108)	32%	(97)	

Values are percent (n)

^*^Defined according to land ownership as landless (0 ha), small/marginal farms (0.01-2.00 ha), medium farms (2.01-4.00 ha), and large farms (>4.00 ha)

^†^P value from chi-square test comparing characteristics across farm sizes

^‡^Other crops included (in order of frequency reported): fruit, mustard, millet, cotton, groundnut, sugarcane, sesame, flowers, fodder, and silk

^§^Change relative to previous harvest of the same crop – Rabi 2019 or, for vegetables, January/February 2020

^#^This question was added to the survey partway through data collection and therefore is missing for 47.1% of respondents

A majority of participants had harvested wheat in the past month (60%) followed by vegetables (15%), pulses (4%), rice paddy (3%), and maize (3%) (Table 2). In terms of what was done with the harvested crop for those who did harvest, 2% reported that their harvest was wasted because they could not sell it, and, in a small number of cases, due to inclement weather. A majority of participants, however, were able to sell their crops (44%) or stored them (39%); though many who stored their crops did so because of lockdown-related issues (e.g., market price was too low) (Fig. 2b). An additional 12% were still trying to sell their crops. Landless and small/marginal farmers were significantly less likely to sell their crops as compared to medium and large farmers. In terms of state-wise comparisons, farmers in Bihar and Rajasthan were least likely to have sold their crops and farmers in Andhra Pradesh, Haryana, and Punjab most likely (**Supplementary Table** **3**). Crop-wise comparisons indicated that those harvesting vegetables were most likely to be still trying to sell them and most likely to have experienced waste – vegetables accounted for nearly all of the wasted crops in this sample (**Supplementary Table** **4**). Differences across caste groups were also observed: those self-reporting Other/No answer (versus Scheduled Caste/Tribe or Other Backward Caste) were significantly more likely to be medium or large farmers, to have harvested in the past month, and to have sold their harvest (**Supplementary Table** **5**). They were also less likely to report a decrease in land harvested or higher costs to harvest, but more likely to report a higher cost of transport.

In terms of changes in harvest since last season, 13% of participants reported a decline in the land harvested, and landless and small/marginal farmers were significantly more likely to report declines (17–20% compared to 10% among large farmers) (Table 2). In terms of yields from harvested land, 62% reported a yield loss. About 13–14% cited labor shortages, lack of storage facilities, and lack of transport as the underlying reasons for their yield loss, which compounded pre-existing weather- and pest-related risks (Fig. 2c). Over half of participants (53%) reported that the cost to harvest was higher as compared to last season (Table 2). In a subset of participants (*n* = 180) we also asked why the cost was higher. The most frequently reported reasons were higher cost of labor (60%) and higher cost of machinery (47%). Among farmers who were able to sell their harvest in the past month, 36% reported transport costs of 0 INR ($0) compared to 61% who said the same transport would cost 0 INR ($0) in January/February 2020, suggesting that more farmers have had to pay for transport during the past month as compared to just before the COVID-19 lockdown. Over half (55%) of farmers reported that the lockdown has impacted their ability to prepare for the upcoming sowing season. The reasons reported were as follows: cannot afford inputs or input prices too high (34%), shortage of labor (22%), inputs (especially seeds and fertilizer) not available (20%), and high cost of labor (4%).

Among those aware of government support measures for agriculture during the lockdown (*n* = 33; 9%), only 47% said that they had benefited from them. More than one-third of participants had received a lockdown-specific cash transfer from the government (Table 3). Those who did not receive a cash transfer were significantly (*p* = 0.001) more likely to sell their crop (47% versus 37% among those who received a cash transfer) or be trying to sell it presently (13% versus 10% among those who received a cash transfer). Those who received a cash transfer were significantly (*p* < 0.0001) less likely to be concerned about the upcoming sowing season (46% versus 61% among those who did not receive a cash transfer).

**Table 3:** Government support and livelihoods in participants from agricultural households across 12 states and 200 districts in India during the national COVID-19 lockdown, according to farm size

Characteristic	Total (*n*=1437)		Farm Size^*^								*P* value^†^
			Landless (*n*=88)		Small/Marginal (*n*=722)		Medium (*n*=286)		Large (*n*=318)		
Aware of government support measures for agriculture during lockdown											
Yes	9%	(33)	13%	(1)	9%	(12)	10%	(10)	8%	(10)	0.91
No	91%	(348)	88%	(7)	91%	(127)	90%	(90)	92%	(120)	
Received cash transfer from government since the lockdown											
Yes	37%	(523)	51%	(43)	43%	(307)	37%	(103)	21%	(66)	<0.0001
No	63%	(891)	49%	(42)	57%	(407)	63%	(178)	79%	(249)	
Received extra food rations											
Yes	51%	(718)	73%	(62)	60%	(428)	44%	(123)	29%	(92)	<0.0001
No	49%	(696)	27%	(23)	40%	(287)	56%	(158)	71%	(222)	
Who gave food rations											
Government	98%	(701)	100%	(62)	99%	(421)	96%	(117)	97%	(88)	0.04
NGO	1%	(8)	0%	(0)	1%	(4)	2%	(2)	2%	(2)	
Community members	1%	(4)	0%	(0)	0%	(0)	2%	(3)	1%	(1)	
Anyone in household work for wages											
Yes	32%	(450)	81%	(70)	37%	(256)	21%	(56)	17%	(54)	<0.0001
No	68%	(935)	19%	(16)	63%	(438)	79%	(217)	83%	(258)	
How many in household work for wages	1.92	(1.15)	1.95	(1.27)	1.90	(1.14)	1.84	(0.88)	2.08	(1.11)	0.70
Change in total household wages since lockdown among wage-workers											
Higher	7%	(31)	3%	(2)	10%	(25)	5%	(3)	2%	(1)	0.05
Lower	79%	(345)	87%	(52)	78%	(198)	76%	(42)	74%	(39)	
Same	14%	(60)	10%	(6)	12%	(31)	18%	(10)	25%	(13)	
Change in wages since lockdown, %	−76.08	(42.14)	−80.32	(33.60)	−76.53	(43.83)	−65.30	(49.88)	−74.46	(37.39)	0.28
Wages declined by 50% or more since lockdown											
Yes	94%	(324)	92%	(48)	94%	(186)	90%	(38)	97%	(38)	0.61
No	6%	(21)	8%	(4)	6%	(12)	10%	(4)	3%	(1)	
Anyone in household currently outside village for work											
Yes	20%	(87)	10%	(6)	22%	(57)	18%	(10)	24%	(12)	0.18
No	80%	(346)	90%	(53)	78%	(197)	82%	(45)	76%	(39)	
How many outside village	1.41	(0.93)	1.50	(1.22)	1.30	(0.82)	1.80	(1.03)	1.67	(1.23)	0.33
Unable to migrate for work due to lockdown											
Yes	30%	(130)	36%	(21)	32%	(80)	25%	(14)	16%	(8)	0.08
No	70%	(302)	64%	(38)	68%	(173)	75%	(41)	84%	(43)	
How many unable to migrate	1.55	(1.11)	1.29	(0.56)	1.59	(1.21)	2.00	(1.36)	1.25	(0.89)	0.26
Own livestock											
Yes	77%	(1091)	49%	(42)	77%	(557)	77%	(217)	83%	(263)	<0.0001
No	23%	(331)	51%	(43)	23%	(163)	23%	(64)	17%	(53)	
Own cow/ buffalo/ ox/ bull, % yes	94%	(1022)	88%	(37)	93%	(516)	95%	(206)	95%	(251)	0.16
Own poultry, % yes	9%	(99)	2%	(1)	13%	(70)	6%	(14)	5%	(14)	0.001
Own goat/ sheep, % yes	16%	(178)	17%	(7)	19%	(108)	15%	(32)	11%	(30)	0.03
Income from livestock in past month											
Yes	28%	(306)	21%	(9)	25%	(139)	34%	(74)	31%	(81)	0.04
No	72%	(785)	79%	(33)	75%	(418)	66%	(143)	69%	(182)	
Income from livestock in Jan/Feb 2020											
Yes	38%	(411)	21%	(9)	35%	(195)	45%	(97)	40%	(106)	0.008
No	62%	(680)	79%	(33)	65%	(362)	55%	(120)	60%	(157)	
Decline in income from livestock since Jan/Feb 2020											
Yes	49%	(138)	60%	(3)	45%	(58)	51%	(36)	53%	(39)	0.66
No	51%	(142)	40%	(2)	55%	(70)	49%	(35)	47%	(34)	
Change in livestock income, %	−12.85	(33.03)	−41.07	(39.82)	−8.75	(41.22)	−15.69	(21.40)	−15.57	(24.66)	0.12
Catch fish											
Yes	6%	(83)	0%	(0)	10%	(71)	2%	(5)	2%	(7)	<0.0001
No	94%	(1339)	100%	(85)	90%	(649)	98%	(276)	98%	(309)	
Income from fishing in past month											
Yes	8%	(8)	0%	(0)	10%	(7)	0%	(0)	11%	(1)	0.70
No	92%	(90)	100%	(3)	90%	(66)	100%	(10)	89%	(8)	
Income from fishing in Jan/Feb 2020											
Yes	18%	(18)	0%	(0)	25%	(18)	0%	(0)	0%	(0)	0.08
No	82%	(80)	100%	(3)	75%	(55)	100%	(10)	100%	(9)	

Values are percent (n) or mean (SD)

^*^Defined according to land ownership as landless (0 ha), small/marginal farms (0.01-2.00 ha), medium farms (2.01-4.00 ha), and large farms (>4.00 ha)

^†^P value from chi-square test (binary and categorical variables) or analysis of variance (continuous variables) comparing characteristics across farm sizes

One-third of participants reported household members working for wage-income (Table 3). Of these households, 20% reported members who have currently migrated for work and 30% reported members who are currently unable to migrate for work due to the lockdown. Among those reporting wage-income, 79% had lower wages during the lockdown as compared to this time last year. A majority of participants owned livestock (77%) with 94% owning cattle and 9% owning poultry. Most (72%) used livestock products exclusively for home consumption. Of those earning an income, 49% reported a lower income in the past month as compared to January/February 2020. Those who had a lower livestock income in the past month were significantly (*p* < 0.0001) more likely to be concerned about the upcoming sowing season (68% versus 45% among those who did not have a lower livestock income in the past month). There was no significant association between decline in livestock income and what was done with the harvest in the past month (*p* = 0.20).

Landless farmers were 10 times more likely to skip a meal and small/marginal farmers nearly 3 times more likely as compared to large farmers (Table 4). A majority reported receiving extra food rations from the government (51% overall and 73% among landless farmers). All farmers reported consuming grains in the past week, 92% reported consuming pulses, 96% reported consuming vegetables, 86% reported consuming dairy, and 83% reported consuming potatoes. Very few reported eating fried food, sweets, or drinking sugary drinks (~8–14%). Landless farmers were significantly less likely to consume potatoes, pulses, and vegetables. Landless and small/marginal farmers were significantly less likely to consume fruit and dairy. Large farmers had the highest intake of nuts and fried foods, and medium farmers had the highest intake of meat, poultry, and eggs. Dietary diversity was, on average, 2.34 nutrient-dense items out of 8 consumed daily ranging from 2.20 among landless farmers to 2.62 among large farmers.

**Table 4:** Food insecurity and dietary diversity in participants from agricultural households across 12 states and 200 districts in India during the national COVID-19 lockdown, according to farm size

Characteristic	Total (*n*=1437)		Farm Size^*^								*P* value^†^
			Landless (*n*=88)		Small/Marginal (*n*=722)		Medium (*n*=286)		Large (*n*=318)		
Worry about food in past month											
Yes	30%	(426)	52%	(44)	36%	(255)	25%	(70)	14%	(44)	<0.0001
No	70%	(994)	48%	(41)	64%	(463)	75%	(211)	86%	(272)	
Skipped a meal in past month											
Yes	5%	(66)	18%	(15)	5%	(33)	2%	(7)	2%	(7)	<0.0001
No	95%	(1355)	82%	(70)	95%	(686)	98%	(274)	98%	(309)	
Went without eating for a whole day in past month											
Yes	1%	(20)	7%	(6)	1%	(8)	1%	(2)	1%	(2)	<0.0001
No	99%	(1400)	93%	(79)	99%	(710)	99%	(279)	99%	(314)	
Food consumption											
Grains, % yes	100%	(1413)	100%	(85)	100%	(714)	100%	(280)	100%	(315)	–
Grains, days per week	6.83	(0.50)	6.98	(0.22)	6.81	(0.53)	6.79	(0.53)	6.87	(0.45)	0.006
Potatoes, % yes	83%	(1173)	73%	(60)	82%	(582)	85%	(238)	88%	(278)	0.003
Potatoes, days per week	2.55	(2.31)	2.61	(2.43)	2.67	(2.40)	2.38	(2.25)	2.43	(2.14)	0.22
Pulses, % yes	92%	(1296)	80%	(67)	93%	(661)	91%	(254)	96%	(301)	<0.0001
Pulses, days per week	4.15	(2.37)	3.17	(2.70)	4.19	(2.31)	4.32	(2.41)	4.22	(2.28)	0.0009
Nuts, % yes	16%	(226)	14%	(12)	12%	(84)	16%	(44)	27%	(85)	<0.0001
Nuts, days per week	0.38	(1.12)	0.26	(0.89)	0.26	(0.95)	0.32	(0.93)	0.72	(1.51)	<0.0001
Vegetables, % yes	96%	(1351)	89%	(75)	96%	(686)	96%	(268)	97%	(306)	0.01
Vegetables, days per week	5.00	(2.13)	4.39	(2.45)	5.13	(2.11)	4.99	(2.08)	4.95	(2.05)	0.02
Fruit, % yes	49%	(696)	40%	(34)	46%	(330)	56%	(156)	53%	(168)	0.006
Fruit, days per week	1.45	(2.02)	0.76	(1.10)	1.25	(1.86)	1.71	(2.14)	1.87	(2.33)	<0.0001
Meat, % yes	11%	(154)	11%	(9)	9%	(62)	17%	(48)	11%	(35)	0.002
Meat, days per week	0.12	(0.45)	0.11	(0.31)	0.10	(0.39)	0.20	(0.57)	0.13	(0.50)	0.02
Poultry, % yes	17%	(240)	8%	(7)	18%	(129)	23%	(65)	12%	(38)	<0.0001
Poultry, days per week	0.21	(0.55)	0.09	(0.33)	0.21	(0.48)	0.28	(0.58)	0.20	(0.70)	0.04
Fish, % yes	13%	(188)	0%	(0)	18%	(128)	12%	(32)	8%	(25)	<0.0001
Fish, days per week	0.23	(0.69)	0	(0)	0.34	(0.85)	0.15	(0.46)	0.13	(0.51)	<0.0001
Dairy, % yes	86%	(1207)	67%	(57)	82%	(586)	92%	(256)	94%	(297)	<0.0001
Dairy, days per week	5.44	(2.57)	4.20	(3.30)	5.02	(2.72)	6.00	(2.12)	6.35	(1.76)	<0.0001
Eggs, % yes	33%	(455)	24%	(20)	36%	(256)	37%	(103)	22%	(70)	<0.0001
Eggs, days per week	0.65	(1.17)	0.51	(1.06)	0.70	(1.17)	0.72	(1.18)	0.50	(1.12)	0.03
Fried foods, % yes	9%	(132)	2%	(2)	9%	(63)	8%	(21)	14%	(45)	0.002
Fried foods, days per week	0.17	(0.65)	0.04	(0.24)	0.14	(0.59)	0.15	(0.63)	0.29	(0.83)	0.001
Sweets, % yes	14%	(196)	18%	(15)	12%	(86)	13%	(36)	19%	(59)	0.03
Sweets, days per week	0.40	(1.32)	0.62	(1.73)	0.31	(1.15)	0.43	(1.41)	0.55	(1.48)	0.03
Sugary drinks, % yes	8%	(107)	13%	(11)	7%	(51)	9%	(25)	6%	(19)	0.14
Sugary drinks, days per week	0.16	(0.66)	0.22	(0.78)	0.16	(0.66)	0.18	(0.61)	0.15	(0.67)	0.78
Dietary diversity^‡^	2.34	(1.17)	2.20	(1.00)	2.23	(1.13)	2.39	(1.24)	2.62	(1.19)	<0.0001

Values are percent (n) or mean (SD)

^*^Defined according to land ownership as landless (0 ha), small/marginal farms (0.01-2.00 ha), medium farms (2.01-4.00 ha), and large farms (>4.00 ha)

^†^P value from chi-square test (binary and categorical variables) or analysis of variance (continuous variables) comparing characteristics across farm sizes

^‡^Those who consumed a food group every day in the past week were assigned a value of “1” and those who did not were assigned a value of “0.” Values were then summed across eight food groups (starchy staples [rice, wheat, and potatoes], pulses, nuts, vegetables, fruits, dairy, eggs, and fleshy foods [meat, poultry, and fish]) such that the dietary diversity score ranged from 0 to 8 with 8 representing maximum dietary diversity

## Discussion

In this sample, covering 200 districts in India, farmers’ primarily reported difficulty in selling their crops and livestock products during the COVID-19 lockdown, higher transport costs, and drastically lower daily wages compared to before the lockdown—with wages declining, on average, by nearly 80% as compared to this time last year. This has left many without the necessary cash to purchase inputs for the upcoming sowing season. The situation has been compounded by weather-induced harvest disruptions and yield losses. One in three farmers was worried about running out of food, and landless farmers were substantially more vulnerable to severe forms of food insecurity, e.g., skipping meals and going a whole day without eating. In this study, those who received a cash transfer were significantly less likely to be concerned about the upcoming sowing season, providing an early indication that the scheme helped recipients to cope with the situation. Our findings also suggest that the PDS has been largely effective—a majority of respondents reported receiving extra food rations. In sum, findings reveal that COVID-19 has exacerbated pre-existing issues in the agri-supply chain in India ultimately resulting in increased distress among already vulnerable agricultural households.

While we cannot conclusively state that these observations are the result of the COVID-19 lockdown, the strength of the effect, the consistency of the effect across states, the temporality of the effect, and the clear underlying mechanism through which the lockdown could affect these outcomes, together lend support to causality (Rothman and Greenland 2005). In addition, our sample of 1437 respondents cannot be considered a random or representative sample, particularly for a country as large and diverse as India. Nearly all participants were male and our sample was younger and had a higher education as compared to nationally representative samples of agricultural households in India. Land ownership was, on average, 3.13 ha, which is nearly 3 times that of the national average land-holding size of 1.08 ha (Ministry of Agriculture and Farmers Welfare 2018). Only 6% of participants were landless farmers (agri-workers), and so readers should be particularly cautious when generalizing findings for this especially vulnerable population.

Eleven percent of farmers reported not harvesting a crop in the past month, and 24% of these cited the lockdown as the underlying reason. This is a much lower percent than that reported in a previous study, which found 34% of farmers were unable to harvest their crop (Azim Premji University 2020). Similarly, a survey of 5022 farmers across 23 states reported 41% could not harvest in 2020 (Gaon Connection 2021). About one-quarter of the farmers sampled in our study were outside their harvest season (e.g., they had already harvested before the lockdown or were planning to harvest after the monsoon). Together, those who did not harvest and those who were outside their harvest season in our sample was about 36%, quite similar to previous study estimates. A number of farmers (12%) in our sample reported that they were still trying to sell their harvest, and an additional 21% of farmers had stored their produce due to the lockdown, indicating that a significant share of farmers faced market-related problems in the past month. Disruptions to harvesting were most significant for vegetable farmers in our sample, which is consistent with a previous study across four states, which found 69% of vegetable farmers said prices were too low to continue production (Harris et al. 2020). Moreover, a study in Haryana and Odisha found a majority of farmers (61% and 74%, respectively) stored their harvest because they could not sell it immediately (Ceballos et al. 2020).

Rabi was already underway at the time the lockdown was imposed, which may explain why only a small proportion (13%) of farmers reported that they harvested a smaller amount of land as compared to last season – indeed, a similar percent (16%) reported they harvested a larger amount of land. Our findings based on farmer self-report are similar to those recently reported using satellite remote sensing data: between 2019 and 2020, there was an overall increase in land area cultivated during Rabi for wheat, pulses, and rice, but not for oilseeds (Saxena et al. 2021). However, a majority of farmers also did report lower yields (62%) and higher harvesting costs (53%) compared to their most recent previous harvest of the same crop (e.g., Rabi 2019 for wheat and rice, and January/February 2020 for vegetables), and many (43%) reported higher transport costs compared to what the same transport would have cost in January/February 2020. While weather and pests were cited as leading contributors to yield loss, lockdown-related labor shortages and the high cost of labor were also reported as important contributors to yield loss and increasing harvest costs. With regard to the upcoming sowing season, 55% of respondents reported that the lockdown has impacted their ability to prepare. Most frequently reported barriers to sowing were access and affordability of inputs (particularly seeds and fertilizers) and labor. These findings are consistent with the World Vegetable Center study, which found 61% of vegetable farmers reported a lack of transport, 39% a lack of inputs, and 32% a lack of labor (Harris et al. 2020), and another previous study, which found that 69% of respondents did not have seeds for the upcoming season (Vikas Anvesh Foundation 2020). While concerning, these numbers are difficult to interpret without baseline data. Prior to COVID-19, the cost of cultivation had been steadily increasing: between 1990-91 and 2014–15, the annual increase in the cost of cultivation in India was 2.14% (Srivastava et al. 2017). Increases in labor costs accounted for 53% of recent increases in the cost of cultivation; machinery 16%; and fertilizer, seed, insecticides, and animal labor less than 10% each (Srivastava et al. 2017). At the same time, yields of major food crops (rice, wheat, and maize) have been stagnating (Madhukar et al. 2020). To address these issues prior to COVID-19, government schemes such as PM Kisan – a direct income transfer to farmers – had been adopted. Under that scheme, 6000 INR (~$79) per year in three equal installments is provided to all landholding farmers. In the context of COVID-19, households identified through PM Kisan could be targeted for increased cash-based support and the installment schedule could also be re-evaluated (e.g., providing a single lump sum before the cultivation season rather than three equal installments throughout the year). However, while direct cash transfers offer greater flexibility to farmers, in the specific context of the COVID-19 lockdown, when movements were restricted and some shops did not have inputs stocked, supplying inputs directly may also be required. Indeed, in our sample, while 34% of farmers were concerned about not being able to afford inputs for the upcoming sowing season, 20% were concerned that inputs would not be available.

Nearly 80% of households dependent on wages reported a loss in wage income since the lockdown (94% saw wages drop by more than half). This is even higher than that predicted for Below Poverty Line households in April (61%) in one previous study (Totapally et al. 2020), but similar to that reported in the World Vegetable Center study (90% of farms seeing a drop in income, including 60% seeing a drop by more than half) (Harris et al. 2020). Prior to COVID-19, agricultural wages in India were increasing: average daily wages for farm labor doubled from 83.50 INR (~$1.10) in 1995–96 to 167.50 INR (~$2.22) in 2016–17, and wages for non-farm labor grew by 74% from 140.80 INR (~$1.86) in 1995–96 to 245.00 INR (~$3.24) in 2016–17 (Kumar and Anwer 2020). However, since 2014–15, there has been some stagnation in both farm and non-farm labor wage rates (Kumar and Anwer 2020). Nonetheless, ~80% of wage-earning households reporting a loss in wage income since the lockdown in our sample is unprecedented. In the short-run, this diminishes a household’s ability to meet food and other essential needs. Given that input costs, like the cost of seeds, were a matter of significant concern for surveyed farmers, such a loss in wage-income could translate into a higher dependence on credit for purchasing inputs. The government’s announcement of an additional $5.3 billion of spending under the social security policy known as National Rural Employment Guarantee Scheme (NREGS) (Sharma 2020) may help address this significant dip in wages. NREGS guarantees up to 100 days of unskilled manual labor per year on public works projects. However, even before COVID-19, not all demand for work was met by the scheme (Dutta et al. 2012) and the administrative process for registering for work under the scheme was cumbersome. For example, job cards are required to receive benefits under NREGS. Given the potential positive impact of NREGS on wage income in rural areas, the government should consider removing administrative hurdles such as the requirement for pre-registration for job cards in times of crisis such as COVID-19.

In addition to cultivation and wage labor, livestock is a source of income for agricultural households, making up an estimated 8% of their income according to a national survey conducted in 2016–17 (NABARD 2018). A decline in this income source for half the farmers in our sample is a matter of concern, especially if the overall demand for livestock products continues to be depressed in the coming months. Our findings were similar to a previous study, which reported that 50% of households who sell milk reported a reduction in milk sales as a result of the lockdown (Vikas Anvesh Foundation 2020).

Over half of respondents (51%) reported receiving food-rations above their normal allocation during the lockdown. This may explain why more severe forms of food insecurity (e.g., skipping a meal or going without eating for a whole day) were not prevalent in our sample – just 5% and 1% respectively overall (18% and 7% respectively among landless farmers), which is much lower than 50% reported elsewhere for marginalized communities (Vikas Anvesh Foundation 2020) but similar to a previous study among smallholder farmers in Haryana and Odisha (Ceballos et al. 2020). Almost all of these food rations came from the government. Similar to one previous study, which reported only 6% of respondents received private external support (Totapally et al. 2020), we found very few participants had received support in the form of extra food rations from NGOs or other private external support, but these results are likely to be different in urban areas.

All farmers reported consuming grains in the past week (97% daily), 92% reported consuming pulses (26% daily), 96% reported consuming vegetables (40% daily), and 86% reported consuming dairy (63% daily). Fewer than 2% consumed nuts, fleshy foods, or eggs daily in this sample. A previous study conducted in two states (Gujarat and Haryana) before COVID-19, around the same time of year (May to June 2017), found similar rates of consumption of grains (100% daily), but higher rates of consumption of dairy (94–95% daily), pulses (57–58% daily), vegetables (64–65% daily), and fruit (42–43% daily) (Singh et al. 2020). They also had a higher mean dietary diversity score: ~4 out of 10 (Singh et al. 2020) compared to 2.34 out of 8 in our sample. Unlike most other food groups (e.g., pulses, nuts, vegetables, and fruits), there was not a clear positive gradient between animal-source food consumption and farm size, an important indicator of socioeconomic status in this context. Medium-sized farmers were the most likely to consume meat, and small/marginal and medium-sized farmers were the most likely to consume poultry and eggs. Small/marginal farmers were about twice as likely to consume fish. We have previously observed similar associations in representative samples of Chennai and Delhi wherein the lowest income households and those experiencing food insecurity were more likely to consume meat (Rautela et al. 2020). With the exception of sugary drinks, of which there were very few consumers, there was higher consumption of unhealthy foods including fried foods and sweets among medium and large farmers. In the context of acute food crises, the PDS is clearly an effective approach for getting food to those who most need it – as evidenced by the fact that about half of participants in our sample reported receiving extra food rations. Diversifying the PDS system by including fresh fruits and vegetables, dairy, and eggs, could help improve dietary diversity and prevent largescale waste of these nutrient-dense, perishable products. However, long-term strategies must strengthen food supply chains.

In order to prevent a worsening of food insecurity, the government has announced a few measures to strengthen local food production (The Wire Staff 2020). Such activities include, for example, marketing co-ops, which many civil society organizations and government schemes were promoting prior to the lockdown. However, at the same time, it should be recognized that a majority of food consumed in India before the crisis was purchased. The National Sample Survey Office (2011–2012) estimates that 84% of food consumed by rural households (in value) is purchased from the market (Ministry of Statistics and Programme Implementation 2013). The private sector sells 95% of all purchased food, while only 5% (largely rice and wheat) comes from the government via the PDS (Reardon et al. 2020). Therefore, major efforts should be made to sustain and strengthen food supply chains as (Reardon et al. 2020) found that the government of India food distribution cannot replace even a tenth of the market. Direct income support to consumers to purchase food could help stimulate demand. In addition, (Reardon et al. 2020) proposed government-supported cash-for-work schemes to employ migrant workers who have lost jobs in the distribution of emergency food rations, and upgrading wholesale and wet markets to improve occupational safety, hygiene, and prevent food waste (Reardon et al. 2020). This would not only address part of the employment crisis but also strengthen the food supply chain (Reardon et al. 2020).

Outside of India, to the best of our knowledge, to date, very few studies have systematically evaluated COVID-19-related impacts on agricultural production, livelihoods, and food security in rural areas. One of the first studies, conducted in China via phone interview, found that 63% of the 726 villagers surveyed reported that the prices of foodstuffs were higher than in 2019 (Rozelle et al. 2020). Although the majority of respondents said fruit, vegetables, and grains were all available, nearly half said the quality of their diets fell. However, they did not explicitly evaluate impacts on agricultural production. We were able to identify several other surveys conducted on this topic in the context of COVID-19 in low- and middle-income countries – many were Working Papers from the International Food Policy Research Institute (IFPRI) (**Supplementary Table** **6**). Overall, these studies, similar to our study in India, suggest that the impacts on agricultural production have been slight whereas impacts on livelihoods have been quite severe. As a result, food insecurity and diet quality have worsened, but not to an alarming degree due to coping strategies such as increasing consumption of cheaper, starchy staples, as well as government support. In particular, several studies found that farmers in particular fared better in terms of food insecurity than other populations (Aggarwal et al. 2020; Headey et al. 2020; Kansiime et al. 2021). The FAO (Torero 2020), the World Food Programme (Husain et al. 2020), and IFPRI (Swinnen 2020) have commented on potential impacts of the COVID-19 pandemic and lockdown based on previous research conducted during the 2014 Ebola outbreak (for example, citing that 46% of farmers in Liberia did not plant due to the Ebola outbreak (Gatiso et al. 2018)) or the 2007–08 food crisis (Swinnen 2020), but more COVID-19-specific, empirical research is needed.

This study is not without limitations. While we demonstrated the feasibility of collecting timely, policy-relevant information in the midst of a national lockdown using pre-existing networks of farmers and phone survey interviews, this approach inherently only reaches those who have a phone and network coverage. Many vulnerable populations, made even more vulnerable by losing wages, may have owned phones, but could not afford to purchase minutes, and so we were unable to reach them. And, as mentioned previously, the sample largely consisted of male, land-owning farmers. The most recent Agriculture Census (2015–16) showed that 86% of farmers in India are small/marginal (Department of Agriculture 2020) compared to 51% in our sample. Official estimates of the proportion of farmers that are female are likely grossly underestimated because they are based on land ownership. The same Agriculture Census reported that 14% of operational holdings were female holdings (Department of Agriculture 2020) compared to 6% in our sample. However, the most recent Census of India (2011), which reported economic activity, found that 39% of those reporting cultivation or agricultural labor as their occupation were women (Office of the Registrar General & Census Commissioner 2011). Another limitation was that we were not able to calculate a binary variable to classify individuals as food insecure or food secure because we only asked three of the eight FIES questions. This change was made based on pilot testing that indicated the food insecurity questions were very sensitive, and so we opted to ask just three questions to ensure data quality and to avoid undue participant discomfort. In addition, phone surveys are more susceptible to participant’s losing focus or dropping off before completion, and so we tried to keep the survey succinct and this precluded our ability to ask in-depth queries about certain aspects of the outcomes of interest; e.g., portion sizes for foods. Surveys also inherently rely on participant self-report, which may be biased, particularly in the context of the COVID-19 pandemic. The sequence of questions, interpretation of questions, and translations are critical. We conducted extensive pilot-testing of the survey tool prior to implementation, all enumerators were trained, and we had daily communication with enumerators throughout the collection process such that clarifying questions were answered in real-time. Nonetheless, systematic biases may remain. For example, in under-reporting of food insecurity given that participants expressed some discomfort in being asked these questions. Participants also expressed some frustration regarding the questions on nut and fruit consumption as these are expensive, “special” foods, unaffordable in this context. Finally, the sources of distress were different for farmers in different socio-economic groups, growing different crops, and in different agro-climatic zones. In order to prepare for future pandemics, local, state- and crop-specific studies are needed to inform which strategies will be most effective for mitigating negative impacts of pandemic control measures on agricultural production, livelihoods, and food security identified in this study. Such studies are already being conducted to inform state-wise adaptation strategies for climate change, and similar approaches could be used for pandemic preparedness.

In terms of next steps, we will continue to follow participants to monitor agricultural production, livelihoods, and food security in this population. To date, the focus has been on the lockdown given the relatively low number of COVID-19 cases in India. However, many rural areas in India are in fact peri-urban (Pingali et al. 2019), and so it is conceivable that COVID-19 could spread in agricultural areas with additional adverse effects. In conclusion, our baseline findings confirm that landless and small/marginal farmers are the most vulnerable to lockdown-related disruptions to agriculture and food insecurity, and government efforts to address gaps identified herein should be implemented to avoid further economic and nutritional disparities. Most of the disruptions observed in this study can be planned for to avoid similar disruptions during future lockdowns and pandemics. Specifically, government emergency food rations such as PDS, work guarantee schemes such as NREGS, and supply of agricultural inputs can play an important role if mobility is restricted and supply chains are severely disrupted (making the cash unhelpful in that moment of crisis). However, once the immediate crisis is adverted, there must be a transition from these emergency stopgap measures to concerted efforts to strengthen food supply chains, thus building longer-term resilience for agricultural production, livelihoods, and food security.

## Supplementary Information

ESM 1 (DOCX 44 kb)

ESM 2 (DOCX 74 kb)
