# Supplementary material for: Impact of the COVID-19 pandemic on agricultural production, livelihoods, and food security in India: baseline results of a phone survey
Source: Food Secur. 2021 May 13;13(5):1323–39. doi: 10.1007/s12571-021-01164-w (PMC8116443; doi:10.1007/s12571-021-01164-w)
Supplement: Supplementary file 1 — (DOCX 44 kb) [file 12571_2021_1164_MOESM1_ESM.docx]

| **No.** | **Question** | **Response Categories** | **Response** |
| --- | --- | --- | --- |
| **Demographics and contact information** | | | |
|  | Did participant provide verbal consent to participate? | 1= Yes  0= No (end survey) |  |
|  | How old are you? | Years  *If <18 years of age, end survey.* |  |
|  | Gender of participant | 1= Male  2= Female |  |
|  | Name of participant | Text |  |
|  | State | List 12 states |  |
|  | District | List districts by state |  |
|  | Primary phone number | 10-digit number  *If no change in phone number, enter “same”* |  |
|  | Secondary phone number | 10-digit number  *Leave blank if no secondary phone number* |  |
|  | How many people live in your household? | 1  2  3  4  5  6 or more  Refused |  |
|  | What is your highest level of education?  *Highest level of education of the person you are speaking to*  *Official definition for "literate" is: Can you write your name?* | 1= Illiterate  2= Literate, no formal education  3= Primary school (up to Standard 5)  4= Middle school (Standard 5-8)  5= High school (Standard 10)  6= Higher secondary school (+2)  7= Graduate (BA/BSc/BCom/Diploma)  8= Professional degree/Post graduate |  |
|  | How much land do you own? | number |  |
|  | Units | Don’t own land  1= acres  2= cents  3= guntas  4= bhiga  5= kila  6= biswa  7= kattha  8= marla  9= ghaj  10= mann  11= Other (specify) |  |
|  | Second land ownership value (as needed) | number |  |
|  | Units | 1= acres  2= cents  3= guntas  4= bhiga  5= kila  6= biswa  7= kattha  8= marla  9= ghaj  10= mann  11= Other (specify) |  |

| **Crops harvested** | | | |
| --- | --- | --- | --- |
|  | Did you harvest any crop in the **past month**? | 1= Yes  0= No (answer Q16 then skip to Q52) |  |
|  | If no, why did you not harvest any crop?  *Choose all that apply* | 1= No crop to harvest because they did not plant  2= Crop not ready to harvest yet  3= Not enough crop to harvest due to weather  4= Not enough crop to harvest due to pests  5= High cost of labor  6= Labor not available  7= Machinery not available  8= Market price too low  9= Limited access to cash (e.g. cannot travel to ATMs or banks to take loans)  10= Government did not permit  11= Leasing out my land (skip to Q52)  12= Could not reach farm on time due to lockdown  13= Other (specify) |  |
|  | Which of these is your primary crop?  *“Primary” refers to the crop for which you make the most money*  *Choose one* | 1= Paddy  2= Wheat  3= Maize  4= Mustard  5= Pulse  6= Sesame  7= Groundnut  8= Vegetables  9= Orange  10= Mango  11= Other fruit  12= Sugar cane  13= Millet  13= Other (specify) |  |
|  | How much [PRIMARY CROP NAME] did you harvest in the **past month**?  *If range is given, enter the mid-way number*  *If they are still harvesting, this refers to the total acres harvested till now* | number |  |
|  | Units | 1= acres  2= cents  3= guntas  4= bhiga  5= kila  6= biswa  7= kattha  8= marla  9= ghaj  10= mann  11= Other (specify) |  |
|  | Second land harvested value (as needed) | number |  |
|  | Units | 1= acres  2= cents  3= guntas  4= bhiga  5= kila  6= biswa  7= kattha  8= marla  9= ghaj  10= mann  11= Other (specify) |  |
|  | How much [PRIMARY CROP NAME] did you harvest **last season**?  *For perennial crops, “last season” refers to January/February*  *If they didn’t harvest this crop last season, enter 0*  *If range is given, enter the mid-way number* | number |  |
|  | Units | No harvest last season (skip all “last season” questions)  1= acres  2= cents  3= guntas  4= bhiga  5= kila  6= biswa  7= kattha  8= marla  9= ghaj  10= mann  101= Other (specify) |  |
|  | Second land harvested last season value (as needed) | number |  |
|  | Units | 1= acres  2= cents  3= guntas  4= bhiga  5= kila  6= biswa  7= kattha  8= marla  9= ghaj  10= mann  11= Other (specify) |  |
|  | Was there a difference in the cost to harvest [PRIMARY CROP NAME] compared to last season? | 1= Higher  2= Lower  3= Same (skip to Q28) |  |
|  | Why was it different?  *Choose all that apply* | 1= Labor cost was higher  2= Labor cost was lower (our family harvested)  3= Machine rental cost was higher  4= Diesel cost was higher  5= Other (specify) |  |
|  | What was the average cost to harvest [PRIMARY CROP NAME] in the **past month**?  *If range is given, enter the mid-way number*  *If reported in per hour costs, probe for number of hours per acre* | INR |  |
|  | Units | 1= per acre  2= total  3= other (specify) |  |
|  | What was the average cost in INR to harvest [PRIMARY CROP NAME] **last season**?  *For perennial crops, “last season” refers to January/February*  *If range is given, enter the mid-way number*  *If reported in per hour costs, probe for number of hours per acre* | INR |  |
|  | Units | 1= per acre  2= total  3= other (specify) |  |
|  | Was there a yield loss for [PRIMARY CROP NAME] compared to last season? | 1= Yes  0= No (skip to Q34) |  |
|  | If yes, why was there a yield loss?  *Choose all that apply* | 1= Labor not available  2= Machinery not available  3= Transport to facility not available  4= Drying facility not available  5= Storage facility not available  6= Weather  7= Pests  8= Other (specify) |  |
|  | What was your yield for [PRIMARY CROP NAME] in the **past month**?  *If range is given, enter the mid-way number*  *Enter in kg (1 quintal = 100 kg)* | kg |  |
|  | Units | 1= per acre  2= per week  3= total  4= per mann  5= per harvest  6= per bag (specify kg/bag)  7= per truckload (specify kg/bag)  8= other (specify) |  |
|  | What was your yield for [PRIMARY CROP NAME] **last season**?  *For perennial crops, “last season” refers to January/February*  *If range is given, enter the mid-way number*  *Enter in kg (1 quintal = 100 kg)* | kg |  |
|  | Units | 1= per acre  2= per week  3= total  4= per mann  5= per harvest  6= per bag (specify kg/bag)  7= per truckload (specify kg/bag)  8= other (specify) |  |
|  | What did you do with the [PRIMARY CROP NAME] you harvested in the past month?  *If farmer gives multiple answers, choose what they did with most of the crop* | 1= Sold it (skip to Q40)  2= Stored it (answer Q39-41 then skip to Q52)  3= Trying to sell it (skip to Q52)  4= Not yet decided (skip to Q52)  5= Other (specify) (skip to Q52) |  |
|  | If stored, why did you store it?  *Choose all that apply* | 1= Low market price  2= High transport cost  3= For home consumption (skip to Q52)  4= Government has not permitted yet  5= No gunny bags  6= Other (specify) |  |
|  | What was the total cost of transportation, including labor, for [PRIMARY CROP NAME]?  *If range is given, enter the mid-way point* | INR |  |
|  | What would have been the total cost for the same transportation, including labor, in **January/February**?  *If range is given, enter the mid-way point* | INR |  |
|  | What was the market price for [PRIMARY CROP NAME] in the **past month**?  *If range is given, enter the mid-way point*  *Probe to get per quintal or per kg. Only select per bag or truckload if absolutely necessary* | INR |  |
|  | Units | 1= per quintal  2= per kg  3= per mann  4= per bag  5= per truckload  6= other (specify) |  |
|  | What was the market price for [PRIMARY CROP NAME] **last season**?  *For perennial crops, “last season” refers to January/February*  *If range is given, enter the mid-way point*  *Probe to get per quintal or per kg. Only select per truckload if absolutely necessary* | INR |  |
|  | Units | 1= per quintal  2= per kg  3= per mann  4= per bag  5= per truckload  6= other (specify) |  |
| **Ask for all crops** | | | |
|  | Do you know of any government support measures for agriculture during the coronavirus lockdown? | 1= Yes  0= No (skip to Q49) |  |
|  | If yes, what were they?  *Choose all that apply* | 1= Village procurement center  2= Other (specify) |  |
|  | Were you able to avail the benefits of these measures?  *If not sure/don’t know yet, select “No”* | 1= Yes  0= No |  |
|  | Has the coronavirus lockdown impacted your ability to prepare for the upcoming sowing season?  *If not sure/don’t know yet, select “No”* | 1= Yes  0= No (skip to Q51)  999= Not planning to sow next season (skip to Q51) |  |
|  | If yes, how has it impacted your ability to prepare for the sowing season? | 1= Could not till soil  2= Cannot afford inputs  3= Labor shortages  4= Other (specify) |  |
|  | Were there any other farming-related problems you faced due to the coronavirus lockdown? | Text |  |
| **Wage related** | | | |
|  | Do you or anyone in your household work for wages (day-wages or work-contract based wages)?  *This includes people who migrate for work.* | 1= Yes  0= No (skip to Q60) |  |
|  | How many work for wages?  *This includes the respondent.* | Number |  |
|  | Are you or anyone in your household currently outside of the village for work? | 1= Yes  0= No |  |
|  | How many are currently outside for work?  *This includes the respondent.* | Number |  |
|  | Would you or anyone in your household have migrated for work but couldn’t because of the lockdown? | 1= Yes  0= No |  |
|  | How many would have migrated but couldn’t?  *This includes the respondent.* | Number |  |
| *Start loop for each member working* | | | |
|  | What is the income from wages for this member in the **past month**?  *If range is given, enter the mid-way number* | Total INR per month |  |
|  | What was the income for this member from wages at **this time last year**?  *If range is given, enter the mid-way number* | Total INR per month |  |
| *End loop* | | | |
| **Livestock and fishing related** | | | |
|  | Does your household have livestock? | 1= Yes  0= No (skip to Q66) |  |
|  | What livestock?  *Choose all that apply* | 1= Cows/Buffaloes  2= Poultry  3= Goats/sheep  4= Other (specify) |  |
|  | What was your income from livestock?  *If only for home consumption, enter 0.*  *This is gross income, not net income after subtracting fodder. Total for all livestock.* | INR |  |
|  | Units | Home consumption only  1= per day  2= per week  3= per month  4= per 3 months  5= per 6 months  6= other (specify) |  |
|  | What was your income from livestock in **January/February**?  *If only for home consumption, enter 0.* | INR |  |
|  | Units | Home consumption only  1= per day  2= per week  3= per month  4= per 3 months  5= per 6 months  6= other (specify) |  |
|  | Does anyone in your household catch fish? | 1= Yes  0= No (skip to Q71) |  |
|  | What was your income from fishing?  *If only for home consumption, enter 0.* | INR |  |
|  | Units | Home consumption only  1= per day  2= per week  3= per month  4= per 3 months  5= per 6 months  6= other (specify) |  |
|  | What was your income from fishing in **January/February**?  *If only for home consumption, enter 0.* | INR |  |
|  | Units | Home consumption only  1= per day  2= per week  3= per month  4= per 3 months  5= per 6 months  6= other (specify) |  |

| **Food security and diet**  *In the past month, due to the coronavirus lockdown, was there a time when you or others in your household [read all and record response]:* | | | |
| --- | --- | --- | --- |
|  | Worried you would run out of food | 1= Yes  0= No |  |
|  | Skipped a meal | 1= Yes  0= No (skip to Q74) |  |
|  | Went without eating for a whole day | 1= Yes  0= No |  |
|  | Did you or members of your household receive **extra** food rations or cooked meals in the past month?  *Extra food rations are above and beyond regular monthly entitlements* | 1= Yes  2= No (skip to Q76) |  |
|  | If yes, from whom did you receive the food rations or cooked meals? | 1= Government  2= NGO  3= Other (specify) |  |
|  | Did your household receive cash transfer from the government due to the lockdown in the past month?  *If anyone in the household has received it, select “yes.”* | 1= Yes  0= No  999= Don’t know/not sure yet |  |
| *During the* ***past week****, how often have you consumed [read all and record response]:* | | | |
|  | Grains  Rice, dosa, breads, chapatis, roti, sorghum, millet, pasta | Days per week |  |
|  | Potatoes | Days per week |  |
|  | Pulses (lentils, beans, and peas) | Days per week |  |
|  | Nuts and seeds (cashew, almond) | Days per week |  |
|  | Do you eat meat? | 1= Yes  0= No (skip to Q86)  999= Not appropriate question to ask (skip to Q86) |  |
|  | Meat (mutton) | Days per week |  |
|  | Poultry (chicken) | Days per week |  |
|  | Fish | Days per week |  |
|  | Eggs | Days per week |  |
|  | Dairy  Milk, yogurt, cheese. Does NOT include butter/ghee | Days per week |  |
|  | Vegetables  Tomato, onion, lady finger, carrot, spinach, cauliflower. Does NOT include potatoes | Days per week |  |
|  | Fruits  Mango, papaya, banana, avocado, guava, jackfruit, orange, passion fruit, peach, pineapple, watermelon | Days per week |  |
|  | Fried snacks  Samosa, crisps, French fries, and other fried foods | Days per week |  |
|  | Sweets  Cakes, candies, chocolates, biscuits, ice cream | Days per week |  |
|  | Cold drinks | Days per week |  |
| **Snowball sampling** | | | |
|  | Would it be ok for us to call you back in a month? | 1= Yes  0= No |  |
|  | Do you know of any other farmers who may be willing to participate in this survey?  *This is anyone employed in agriculture, not just land-owning farmers. It could include, for example, farm laborers. It can include women.* | 1= Yes (record details on roster)  0= No (end survey) |  |
