# Supplementary material for: Impact of the COVID-19 pandemic on agricultural production, livelihoods, and food security in India: baseline results of a phone survey
Source: Food Secur. 2021 May 13;13(5):1323–39. doi: 10.1007/s12571-021-01164-w (PMC8116443; doi:10.1007/s12571-021-01164-w)
Supplement: Supplementary file 2 — (DOCX 74 kb) [file 12571_2021_1164_MOESM2_ESM.docx]

| **Supplementary Table 1.** Land conversion factors from hextobinary.com (accessed 17 February 2021). |
| --- |
| 1 hectare equivalent |
| 2.471052 acres |
| 247.105381 cents |
| 98.842153 gunthas |
| 3.954412 bigha (Bihar) |
| 6.177635 bigha (Gujrat) |
| 8.969925 bigha (Madhya Pradesh) |
| 3.953686 bigha (Rajasthan) |
| 3.986633 bigha (Uttar Pradesh) |
| 7.474938 bigha (West Bengal) |
| 2.471054 killa |
| 79.732670 biswa |
| 149.498756 katha |
| 395.368610 marla |
| 11,959.900463 gaj |

| **Supplementary Table 2.** Summary of missing data. | |
| --- | --- |
| **Variable** | **Missing** |
| State | 0% (0) |
| Gender | 0% (0) |
| Age | 0.6% (9) |
| Household size | 0.5% (7) |
| Education attainment | 0.4% (5) |
| Caste | 27.3% (392) |
| Land ownership | 1.6% (23) |
| Harvested in past month | 3.0% (43) |
| Primary crop harvested in past month | 0% (0) |
| What was done with the harvest in past month | 1.1% (10) |
| Change in land harvested | 0% (0) |
| Yield loss | 0.4% (3) |
| Change in cost to harvest | 0% (0) |
| Change in transport cost | 52.6% (465) |
| Lockdown impacted ability to sow for upcoming season | 4.4% (39) |
| Aware of government support measures for agriculture during lockdown | 57.0% (504) |
| Received cash transfer from government since the lockdown | 1.6% (23) |
| Received extra food rations | 1.6% (23) |
| Who gave food rations | 0.7% (5) |
| Anyone in household work for wages | 3.6% (52) |
| How many in household work for wages | 3.6% (16) |
| Change in total household wages since lockdown among wage-workers | 3.1% (14) |
| Percent change in total household wages since lockdown among wage-workers | 11.1% (50)^*^ |
| Wages declined by 50% or more since lockdown | 23.3% (105)^*^ |
| Anyone in household currently outside village for work | 3.8% (17) |
| How many currently outside village for work | 0% (0) |
| Unable to migrate for work due to lockdown | 0% (0) |
| How many unable to migrate for work | 0% (0) |
| Own livestock | 1.0% (15) |
| Own cow/buffalo/oxen/bull | 0% (0) |
| Own poultry | 0% (0) |
| Own goat/sheep | 0% (0) |
| Income from livestock in past month | 0% (0) |
| Income from livestock in January/February | 0% (0) |
| Decline in income from livestock since January/February | 4.8 (14) |
| Percent change livestock income since January/February | 5.1 (15)^*^ |
| Catch fish | 1.0% (15) |
| Income from fishing in past month | 0% (0) |
| Income from fishing in January/February | 0% (0) |
| Worry about food in past month | 1.2% (17) |
| Skipped a meal in past month | 1.1% (16) |
| Went without eating for a whole day in past month | 1.2% (17) |
| Grains | 1.7% (24) |
| Potatoes | 2.0% (29) |
| Pulses | 1.9% (27) |
| Nuts | 2.1% (30) |
| Meat | 1.7% (24) |
| Poultry | 1.7% (24) |
| Fish | 2.8% (40) |
| Dairy | 1.8% (26) |
| Eggs | 2.7% (39) |
| Vegetables | 1.9% (27) |
| Fruit | 1.8% (26) |
| Fried foods | 1.9% (27) |
| Sweets | 1.8% (26) |
| Sugar-sweetened beverages | 1.8% (26) |
| Dietary diversity | 1.7% (25) |
| Values are percent (n).  ^*^Higher percent missing because cannot divide by 0, not because question was not answered. | |

| **Supplementary Table 3.** Agricultural production in participants from agricultural households across 12 states and 200 districts in India during the national COVID-19 lockdown as compared to last season, according to state. | | | | | | | | | | | | | |
| --- | --- | --- | --- | --- | --- | --- | --- | --- | --- | --- | --- | --- | --- |
|  | State | | | | | | | | | | | | P-value^*^ |
|  | Andhra Pradesh  (n=149) | | Bihar  (n=110) | | Gujarat  (n=88) | | Haryana  (n=83) | | Karnataka  (n=100) | | Madhya Pradesh  (n=149) | |  |
| **Farm size** |  |  |  |  |  |  |  |  |  |  |  |  |  |
| Agri-worker | 17% | (25) | 4% | (4) | 1% | (1) | 20% | (16) | 2% | (2) | 13% | (19) | <0.0001 |
| Small and marginal 0-2.00 ha | 43% | (63) | 60% | (66) | 63% | (55) | 31% | (25) | 59% | (58) | 48% | (70) |  |
| Medium 2.01-4.00 ha | 26% | (39) | 16% | (18) | 22% | (19) | 26% | (21) | 20% | (20) | 20% | (29) |  |
| Large >4.00 ha | 14% | (21) | 20% | (22) | 14% | (12) | 23% | (19) | 19% | (19) | 20% | (29) |  |
| **Harvested in past month** | | |  |  |  |  |  |  |  |  |  |  |  |
| Out of season | 27% | (37) | 1% | (1) | 25% | (22) | 0% | (0) | 36% | (36) | 11% | (15) | <0.0001 |
| Yes | 58% | (81) | 97% | (103) | 66% | (57) | 96% | (79) | 43% | (43) | 78% | (102) |  |
| No | 15% | (21) | 2% | (2) | 9% | (8) | 4% | (3) | 20% | (20) | 11% | (14) |  |
| **Primary crop harvested in past month** | | | |  |  |  |  |  |  |  |  |  |  |
| Wheat | 0% | (0) | 83% | (85) | 32% | (18) | 81% | (64) | 0% | (0) | 75% | (77) | <0.0001 |
| Vegetables | 30% | (24) | 2% | (2) | 25% | (14) | 14% | (11) | 37% | (16) | 1% | (1) |  |
| Pulses | 6% | (5) | 3% | (3) | 2% | (1) | 0% | (0) | 7% | (3) | 15% | (15) |  |
| Rice paddy | 19% | (15) | 0% | (0) | 0% | (0) | 0% | (0) | 7% | (3) | 0% | (0) |  |
| Maize | 10% | (8) | 12% | (12) | 2% | (1) | 0% | (0) | 12% | (5) | 1% | (1) |  |
| Other | 36% | (29) | 1% | (1) | 40% | (23) | 5% | (4) | 37% | (16) | 8% | (8) |  |
| **What was done with the harvest in past month** | | | | |  |  |  |  |  |  |  |  |  |
| Sold it | 63% | (50) | 18% | (19) | 26% | (14) | 66% | (52) | 56% | (24) | 27% | (27) | <0.0001 |
| Stored it | 23% | (18) | 71% | (73) | 59% | (32) | 11% | (9) | 21% | (9) | 52% | (52) |  |
| Trying to sell it | 4% | (3) | 0% | (0) | 0% | (0) | 20% | (16) | 14% | (6) | 21% | (21) |  |
| Not yet decided | 3% | (2) | 10% | (10) | 15% | (8) | 1% | (1) | 2% | (1) | 0% | (0) |  |
| Wasted | 9% | (7) | 1% | (1) | 0% | (0) | 1% | (1) | 7% | (3) | 0% | (0) |  |
| **Change in land harvested** | | |  |  |  |  |  |  |  |  |  |  |  |
| Decrease | 10% | (6) | 28% | (24) | 21% | (7) | 10% | (8) | 24% | (8) | 7% | (2) | <0.0001 |
| Increase | 14% | (8) | 22% | (19) | 52% | (17) | 13% | (10) | 9% | (3) | 33% | (10) |  |
| No change | 76% | (44) | 49% | (42) | 27% | (9) | 77% | (59) | 68% | (23) | 60% | (18) |  |
| **Yield loss** |  |  |  |  |  |  |  |  |  |  |  |  |  |
| Yes | 39% | (25) | 81% | (70) | 53% | (17) | 43% | (33) | 41% | (14) | 48% | (14) | <0.0001 |
| No | 61% | (39) | 19% | (16) | 47% | (15) | 57% | (43) | 59% | (20) | 52% | (15) |  |
| **Change in cost to harvest** | | |  |  |  |  |  |  |  |  |  |  |  |
| Higher | 48% | (31) | 79% | (68) | 67% | (22) | 21% | (16) | 69% | (24) | 64% | (21) | <0.0001 |
| Lower | 18% | (12) | 15% | (13) | 18% | (6) | 22% | (17) | 11% | (4) | 3% | (1) |  |
| Same | 34% | (22) | 6% | (5) | 15% | (5) | 57% | (44) | 20% | (7) | 33% | (11) |  |
| **Change in transport cost** | | |  |  |  |  |  |  |  |  |  |  |  |
| Higher | 22% | (14) | 15% | (2) | 75% | (6) | 72% | (21) | 9% | (3) | 38% | (5) | <0.0001 |
| Lower | 0% | (0) | 0% | (0) | 0% | (0) | 7% | (2) | 6% | (2) | 0% | (0) |  |
| Same | 78% | (50) | 85% | (11) | 25% | (2) | 21% | (6) | 85% | (28) | 62% | (8) |  |
| **Lockdown impacted ability to sow for upcoming season**^†^ | | | | | |  |  |  |  |  |  |  |  |
| Yes | 70% | (101) | 86% | (92) | 52% | (43) | 38% | (29) | 71% | (68) | 57% | (80) | <0.0001 |
| No | 30% | (44) | 14% | (15) | 48% | (40) | 62% | (47) | 29% | (28) | 43% | (60) |  |
| Values are percent (n).  ^*^P-value from chi-square test comparing characteristics across states.  ^†^n=66 who responded, “not planning to sow next season” were excluded from this calculation. | | | | | | | | | | | | | |

| **Supplementary Table 3 (continued).** Agricultural production in participants from agricultural households across 12 states and 200 districts in India during the national COVID-19 lockdown as compared to last season, according to state. | | | | | | | | | | | | | |
| --- | --- | --- | --- | --- | --- | --- | --- | --- | --- | --- | --- | --- | --- |
|  | State | | | | | | | | | | | | P-value^*^ |
|  | Maharashtra  (n=54) | | Punjab  (n=161) | | Rajasthan  (n=131) | | Telangana  (n=180) | | Uttar Pradesh  (n=109) | | West Bengal  (n=123) | |  |
| **Farm size** |  |  |  |  |  |  |  |  |  |  |  |  |  |
| Agri-worker | 6% | (3) | (0) | 0% | 3% | (4) | 1% | (1) | 9% | (9) | 3% | (4) | <0.0001 |
| Small and marginal 0-2.00 ha | 46% | (22) | 23% | (37) | 39% | (51) | 56% | (101) | 65% | (66) | 88% | (108) |  |
| Medium 2.01-4.00 ha | 17% | (8) | 23% | (37) | 20% | (26) | 27% | (49) | 14% | (14) | 5% | (6) |  |
| Large >4.00 ha | 31% | (15) | 53% | (85) | 38% | (50) | 16% | (29) | 12% | (12) | 4% | (5) |  |
| **Harvested in past month** | | |  |  |  |  |  |  |  |  |  |  |  |
| Out of season | 43% | (23) | 1% | (2) | 25% | (32) | 69% | (124) | (0) | 0% | 48% | (59) | <0.0001 |
| Yes | 43% | (23) | 95% | (153) | 62% | (79) | 14% | (25) | 87% | (92) | 38% | (47) |  |
| No | 13% | (7) | 4% | (6) | 13% | (17) | 17% | (30) | 13% | (14) | 14% | (17) |  |
| **Primary crop harvested in past month** | | | |  |  |  |  |  |  |  |  |  |  |
| Wheat | 25% | (6) | 95% | (145) | 86% | (68) | 0% | (0) | 75% | (69) | 0% | (0) | <0.0001 |
| Vegetables | 13% | (3) | 3% | (4) | 0% | (0) | 52% | (13) | 9% | (8) | 81% | (38) |  |
| Pulses | 33% | (8) | 0% | (0) | 1% | (1) | 4% | (1) | 0% | (0) | 4% | (2) |  |
| Rice paddy | 0% | (0) | 0% | (0) | 0% | (0) | 28% | (7) | 1% | (1) | 9% | (4) |  |
| Maize | 0% | (0) | 0% | (0) | 0% | (0) | 8% | (2) | 0% | (0) | 2% | (1) |  |
| Other | 29% | (7) | 3% | (4) | 13% | (10) | 8% | (2) | 15% | (14) | 4% | (2) |  |
| **What was done with the harvest in past month** | | | | |  |  |  |  |  |  |  |  |  |
| Sold it | 48% | (11) | 88% | (135) | 10% | (8) | 54% | (13) | 29% | (27) | 19% | (9) | <0.0001 |
| Stored it | 35% | (8) | 10% | (16) | 84% | (66) | 8% | (2) | 60% | (55) | 8% | (4) |  |
| Trying to sell it | 13% | (3) | 2% | (3) | 6% | (5) | 17% | (4) | 10% | (9) | 63% | (30) |  |
| Not yet decided | 0% | (0) | 0% | (0) | 0% | (0) | 17% | (4) | 0% | (0) | 6% | (3) |  |
| Wasted | 4% | (1) | 0% | (0) | 0% | (0) | 4% | (1) | 1% | (1) | 4% | (2) |  |
| **Change in land harvested** | | |  |  |  |  |  |  |  |  |  |  |  |
| Decrease | 10% | (1) | 4% | (6) | 4% | (3) | 18% | (3) | 13% | (11) | 22% | (10) | <0.0001 |
| Increase | 10% | (1) | 3% | (4) | 13% | (9) | 53% | (9) | 15% | (13) | 9% | (4) |  |
| No change | 80% | (8) | 93% | (136) | 82% | (55) | 29% | (5) | 71% | (60) | 69% | (31) |  |
| **Yield loss** |  |  |  |  |  |  |  |  |  |  |  |  |  |
| Yes | 65% | (11) | 67% | (100) | 52% | (37) | 29% | (7) | 73% | (65) | 90% | (43) | <0.0001 |
| No | 35% | (6) | 33% | (50) | 48% | (34) | 71% | (17) | 27% | (24) | 10% | (5) |  |
| **Change in cost to harvest** | | |  |  |  |  |  |  |  |  |  |  |  |
| Higher | 24% | (4) | 47% | (71) | 56% | (40) | 33% | (8) | 47% | (42) | 73% | (35) | <0.0001 |
| Lower | 41% | (7) | 17% | (25) | 24% | (17) | 38% | (9) | 37% | (33) | 15% | (7) |  |
| Same | 35% | (6) | 36% | (54) | 20% | (14) | 29% | (7) | 16% | (14) | 13% | (6) |  |
| **Change in transport cost** | | |  |  |  |  |  |  |  |  |  |  |  |
| Higher | 0% | (0) | 81% | (114) | 4% | (2) | 47% | (7) | 11% | (4) | 17% | (2) | <0.0001 |
| Lower | 0% | (0) | 3% | (4) | 0% | (0) | 7% | (1) | 3% | (1) | 0% | (0) |  |
| Same | 100% | (6) | 16% | (22) | 96% | (49) | 47% | (7) | 86% | (31) | 83% | (10) |  |
| **Lockdown impacted ability to sow for upcoming season**^†^ | | | | | |  |  |  |  |  |  |  |  |
| Yes | 90% | (44) | 86% | (130) | 79% | (87) | 3% | (5) | 42% | (44) | 25% | (29) | <0.0001 |
| No | 10% | (5) | 14% | (22) | 21% | (23) | 97% | (175) | 58% | (61) | 75% | (85) |  |
| Values are percent (n).  ^*^P-value from chi-square test comparing characteristics across states.  ^†^n=66 who responded, “not planning to sow next season” were excluded from this calculation. | | | | | | | | | | | | | |

| **Supplementary Table 4.** Agricultural production in participants from agricultural households across 12 states and 200 districts in India during the national COVID-19 lockdown as compared to last season, according to primary crop harvested in past month. | | | | | | | | | | | |
| --- | --- | --- | --- | --- | --- | --- | --- | --- | --- | --- | --- |
|  | Crop | | | | | | | | | |  |
|  | Wheat  (n=532) | | Vegetables  (n=134) | | Pulses  (n=39) | | Rice paddy  (n=30) | | Maize  (n=30) | | P-value^*^ |
| **What was done with the harvest in past month** | | | | | | | | | | |  |
| Sold it | 44% | (230) | 41% | (54) | 45% | (17) | 57% | (17) | 57% | (17) | <0.0001 |
| Stored it | 47% | (247) | 17% | (22) | 37% | (14) | 33% | (10) | 33% | (10) |  |
| Trying to sell it | 8% | (44) | 29% | (39) | 16% | (6) | 7% | (2) | 7% | (2) |  |
| Not yet decided | 1% | (6) | 5% | (7) | 3% | (1) | 0% | (0) | 0% | (0) |  |
| Wasted | 0% | (1) | 8% | (11) | 0% | (0) | 3% | (1) | 3% | (1) |  |
| **Change in land harvested** | | |  |  |  |  |  |  |  |  |  |
| Decrease | 10% | (45) | 14% | (15) | 13% | (2) | 23% | (6) | 23% | (6) | 0.02 |
| Increase | 14% | (59) | 15% | (16) | 38% | (6) | 23% | (6) | 23% | (6) |  |
| No change | 76% | (326) | 72% | (78) | 50% | (8) | 54% | (14) | 54% | (14) |  |
| **Yield loss** |  |  |  |  |  |  |  |  |  |  |  |
| Yes | 65% | (277) | 60% | (65) | 56% | (9) | 58% | (15) | 58% | (15) | 0.37 |
| No | 35% | (150) | 40% | (44) | 44% | (7) | 42% | (11) | 42% | (11) |  |
| **Change in cost to harvest** | | |  |  |  |  |  |  |  |  |  |
| Higher | 52% | (223) | 54% | (59) | 38% | (6) | 73% | (19) | 73% | (19) | 0.06 |
| Lower | 20% | (87) | 18% | (20) | 44% | (7) | 15% | (4) | 15% | (4) |  |
| Same | 28% | (120) | 28% | (30) | 19% | (3) | 12% | (3) | 12% | (3) |  |
| **Change in transport cost** | | |  |  |  |  |  |  |  |  |  |
| Higher | 58% | (143) | 34% | (19) | 13% | (2) | 15% | (4) | 15% | (4) | <0.0001 |
| Lower | 2% | (5) | 4% | (2) | 0% | (0) | 4% | (1) | 4% | (1) |  |
| Same | 40% | (100) | 63% | (35) | 88% | (14) | 81% | (21) | 81% | (21) |  |
| Values are percent (n).  ^*^P-value from chi-square test comparing characteristics across crops. | | | | | | | | | | | |

| **Supplementary Table 5.** Agricultural production in participants from agricultural households across 12 states and 200 districts in India during the national COVID-19 lockdown as compared to last season, according to caste. | | | | | | | |
| --- | --- | --- | --- | --- | --- | --- | --- |
|  | Caste | | | | | |  |
|  | Scheduled Caste/Tribe  (n=246) | | Other Backward Caste  (n=398) | | Other/No answer  (n=401) | | P-value^*^ |
| **Farm size** |  |  |  |  |  |  |  |
| Agri-worker | 12% | (29) | 3% | (11) | 6% | (24) | <0.0001 |
| Small and marginal 0-2.00 ha | 59% | (142) | 62% | (245) | 39% | (154) |  |
| Medium 2.01-4.00 ha | 17% | (41) | 18% | (69) | 22% | (89) |  |
| Large >4.00 ha | 12% | (29) | 18% | (69) | 33% | (131) |  |
| **Harvested in past month** | | |  |  |  |  |  |
| Out of season | 32% | (77) | 33% | (129) | 16% | (64) | <0.0001 |
| Yes | 53% | (126) | 56% | (221) | 73% | (286) |  |
| No | 14% | (34) | 11% | (42) | 10% | (41) |  |
| **Primary crop harvested in past month** | | | |  |  |  |  |
| Wheat | 61% | (77) | 56% | (124) | 65% | (186) | 0.03 |
| Vegetables | 15% | (19) | 15% | (34) | 16% | (47) |  |
| Pulses | 8% | (10) | 3% | (7) | 3% | (10) |  |
| Rice paddy | 0% | (0) | 5% | (11) | 2% | (6) |  |
| Maize | 4% | (5) | 5% | (10) | 2% | (6) |  |
| Other | 12% | (15) | 16% | (35) | 11% | (31) |  |
| **What was done with the harvest in past month** | | | | |  |  |  |
| Sold it | 27% | (34) | 32% | (71) | 56% | (161) | <0.0001 |
| Stored it | 58% | (73) | 48% | (106) | 24% | (70) |  |
| Trying to sell it | 13% | (16) | 14% | (30) | 15% | (43) |  |
| Not yet decided | 1% | (1) | 4% | (9) | 2% | (6) |  |
| Wasted | 2% | (2) | 1% | (3) | 2% | (6) |  |
| **Change in land harvested** | | |  |  |  |  |  |
| Decrease | 18% | (14) | 18% | (30) | 9% | (21) | 0.01 |
| Increase | 19% | (15) | 18% | (31) | 14% | (34) |  |
| No change | 63% | (50) | 64% | (108) | 77% | (188) |  |
| **Yield loss** |  |  |  |  |  |  |  |
| Yes | 66% | (52) | 65% | (110) | 59% | (143) | 0.36 |
| No | 34% | (27) | 35% | (59) | 41% | (99) |  |
| **Change in cost to harvest** | | |  |  |  |  |  |
| Higher | 58% | (46) | 63% | (106) | 45% | (110) | <0.0001 |
| Lower | 25% | (20) | 24% | (41) | 23% | (56) |  |
| Same | 16% | (13) | 13% | (22) | 32% | (77) |  |
| **Change in transport cost** | | |  |  |  |  |  |
| Higher | 24% | (10) | 30% | (24) | 56% | (89) | <0.0001 |
| Lower | 2% | (1) | 1% | (1) | 1% | (2) |  |
| Same | 73% | (30) | 69% | (56) | 42% | (67) |  |
| **Lockdown impacted ability to sow for upcoming season**^†^ | | | | | |  |  |
| Yes | 54% | (124) | 48% | (186) | 59% | (224) | 0.02 |
| No | 46% | (107) | 52% | (199) | 41% | (157) |  |
| Values are percent (n).  ^*^P-value from chi-square test comparing characteristics across caste group.  ^†^n=66 who responded, “not planning to sow next season” were excluded from this calculation. | | | | | | | |

| **Supplementary Table 6.** Previous surveys in low- and middle-income countries on agriculture, food security, or nutrition in the context of the COVID-19 pandemic. | | | | |
| --- | --- | --- | --- | --- |
| **First Author, Year** | **Setting** | **Sample** | **Time Period** | **Results** |
| Kansiime, 2021 | Kenya and Uganda | n=442 | April 2020 | - Based on FIES, 26% in Kenya and 9% in Uganda were severely food insecure during the COVID-19 lockdown compared to recall of before the pandemic when 6% and 2% were severely food insecure, respectively - Farmers were less likely to experience food insecurity |
| Amare, 2020 | Nigeria | n=1960 | Pre-COVID-19 survey: 2019  COVID-19 survey: April-May 2020 | - 26% reported skipping a meal pre-COVID-19 and 73% post-COVID-19 - 25% reported running out of food pre-COVID-19 and 57% post-COVID-19 - COVID-19 infection rates, restricted household economic activity, and increased local food prices all adversely impacted food security - Bigger impact on poorer households, households with school-age children, and those living in conflict-affected areas |
| Akinleye, 2020 | Nigeria | n=813 | During lockdown (dates not specified) | - Food insecurity was significantly associated with increases in prices, fall in income, and adverse financial well-being |
| Aggarwal, 2020 | Liberia and Malawi | n=1,189 | First wave: early 2019  Second wave: early 2020 to August 2020 | - 44% in Liberia and 40% in Malawi reported skilling a meal - 23% in Liberia and 27% in Malawi reported going a whole day without eating food - No evidence of decline in food security, possibly because most of the sample relied on subsistence farming - Cash transfers improved dietary quality and quantity |
| Hirvonen, May 2020 | Ethiopia | n=600 | May 2020 + follow-up in June, July and August | - Worsened food security compared to before the pandemic (Jan-Feb 2020) - Lower consumption of expensive but nutritionally beneficial food like meat, fruits and dairy products |
| De Brauw, July 2020 | Ethiopia | n=577 to n=930 | In-person surveys: August to September 2019  In-person follow-up 1: January to February 2020  Phone follow-up 2: May to August 2020 | - Compared to September 2019 food consumption and household dietary diversity are unchanged - Found some changes in composition of food consumption but not related to shocks - Food value chains have been resilient to pandemic shocks |
| Elsahoryia, 2020 | Jordan | n=3129 | First four weeks of the quarantine (dates not specified) | - Based on FIES, 23% were severely food insecure and 36% were moderately food insecure - Moderate food insecurity was associated with monthly income per capita below the poverty line and number of family members - Severe food insecurity was associated with younger age and living in a rented house |
| Ruszczyk, 2020 | Bangladesh | n=201 | First wave: September to October 2019  Second wave: May to July 2020 | - Most respondents reported a significant or complete loss of income, which affected both quantity and quality of food - Coping strategies: relying on inexpensive starchy staples, increased share of expenditure allocated to food, taking out loans, accessing food relief - Disproportionate effect on low-income households |
| Headley, 2020 | Myanmar | n=2017 | June to July 2020 | - 6.6% reported skipping a meal and 1.1% reported going a whole day without eating food - Farmers had the lowest prevalence of food insecurity as compared to unskilled laborers, skilled laborers, salaried, trade/retail, and other livelihoods |
